# Four New Polyprenylated Acylphloroglucinols from *Hypericum perforatum* L.

**DOI:** 10.3390/molecules29081756

**Published:** 2024-04-12

**Authors:** Xiaoying Wang, Wuyang Liu, Sheng Chen, Yueshan Gao, Junmian Tian, Jinming Gao

**Affiliations:** 1 Shaanxi Key Laboratory of Natural Products & Chemical Biology, College of Chemistry & Pharmacy, Northwest A&F University, Yangling 712100, China; wxy19820924@163.com (X.W.); liuwuyang94@nwafu.edu.cn (W.L.); zoyochen@nwafu.edu.cn (S.C.); 17860631668@163.com (Y.G.); 2 Shaanxi Jiahe Phytochem Co., Ltd., Xi’an 710077, China

**Keywords:** *Hypericum perforatum* L., meroterpenoids, polyprenylated acylphloroglucinols, anti-neuroinflammatory

## Abstract

Hyperforatums A–D (**1**–**4**), four new polyprenylated acylphloroglucinols, together with 13 known compounds were isolated and identified from the aerial parts of *Hypericum perforatum* L. (St. John’s wort). Their structures were confirmed with a comprehensive analysis comprising spectroscopic methods, including 1D and 2D NMR, HRESIMS, and electronic circular dichroism (ECD) calculations. Hyperforatum A featured an unusual chromene-1,4-dione bicyclic system, and hyperforatums B and C were two rare monocyclic PPAPs with five-membered furanone cores. Compound **1** exhibited a moderate inhibition effect on NO production in BV-2 microglial cells stimulated by LPS.

## 1. Introduction

The genus *Hypericum* is a large family, consisting of approximately 500 species [1]. Plants of this genus are widely distributed throughout the world and some of them are used as folk medicinal herbs [2]. *H. perforatum* (St. John’s wort) is extensively used to treat mild to moderate mental depression in many countries [3]. In addition, the extracts of *H. perforatum* showed anti-neurodegenerative disease, antitumor, and antimicrobial activities [4,5,6]. Chemical researches studying this plant revealed the presence of diverse PPAPs [7,8,9], flavonoids [10], phenolic acids, and so on [11]. Now, more than 1100 polycyclic polyprenylated acylphloroglucinols (PPAPs) have been isolated and identified from the genus *Hypericum* [12], but complex and novel carbon skeletons of PPAPs are consistently found from this plant, for example, hyperfols A and B [9], hyperforen A [13]**,** hyperforones A–J [14]. Moreover, these PPAPs demonstrated significant neuroprotective effects, especially against Alzheimer’s disease. Thus, the discovery of intricate PPAPs is essential as they are the leading compounds for the treatment of Alzheimer’s disease.

**Figure 1:**
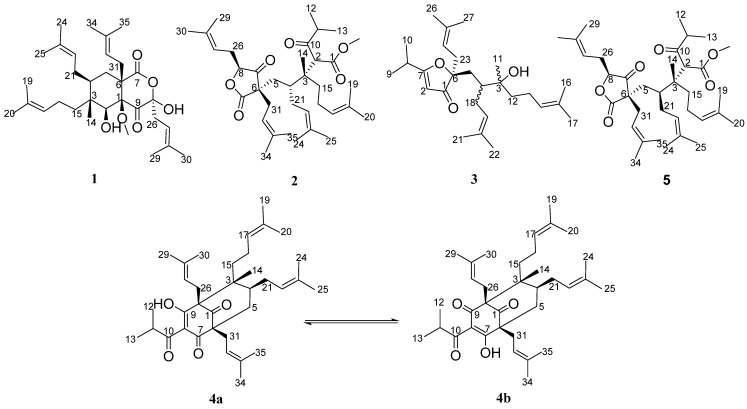
Chemical structures of compounds **1**–**4**.

As a part of our systematic investigation for bioactive PPAPs and terpenoids from genus *Hypericum* plants [15,16], compounds **1**–**17** were obtained and characterized from this plant, including 4 previously undescribed PPAPs, namely hyperforatums A–D (**1**–**4**) (Figure 1), as well as 13 known compounds, a PPAP (**5**), 4 triterpenoids (**6**–**9**), a flavonoid (**10**), a vitamin E derivative (**11**), a diterpenoid (**12**), 3 sesquiterpenoids (**13**, **15**, **16**), a coumarin (**14**), and a dihydroactinidiolide (**17**).

## 2. Results and Discussion

Hyperforatum A (**1**) was obtained as a colorless oil. Its molecular formula was deduced as C_32_H_50_O_6_ based on the ^13^C NMR spectrum and HRESIMS data (*m/z*: [M – H_2_O + H]^+^ calcd. 513.3574; found 513.3573), corresponding to 8 degrees of unsaturation (Figure S8). The ^1^H NMR spectrum of compound **1** displayed characteristic signals for four olefinic protons (*δ*_H_ 5.23, 2H, overlap; 5.05, 2H, overlap) and nine methyls (*δ*_H_ 0.91–1.71, s). Further analysis of its ^13^C NMR and DEPT spectra indicated 32 carbons attributable to 9 methyls, 6 methylenes, 6 methines, 1 methoxyl, and 10 quaternary carbons. The HRESIMS and NMR data revealed that compound **1** should be a bicyclic-type PPAP (Figures S1–S3).

**Figure 2:**
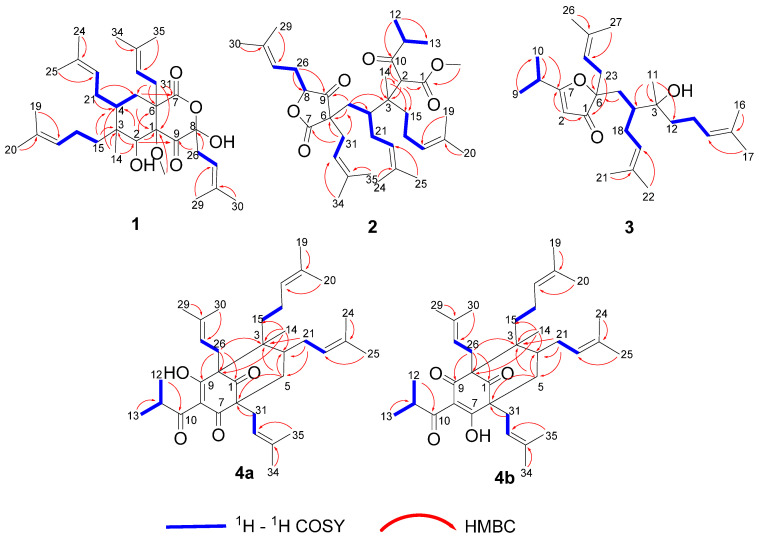
^1^H-^1^H COSY and key HMBC correlations of compounds **1**–**4**.

The planar structure of compound **1** was established by interpreting its 2D NMR data (Figure 2). The HMBC cross peaks from H-2 to C-1/C-3/C-4/C-6/C-9, from H-14 to C-2/C-3/C-4, from H_2_-31 to C-1/C-5/C-6/C-7, as well as the characteristic quaternary carbons (*δ*c 205.1, 180.3, 97.7) confirmed the bicyclic core with a methyl (C-14) and an isoprenyl fragment attached at C-3 and C-6, respectively. Two isoprenyl groups were connected to C-3 and C-4, which were established by the HMBC cross peaks from H_3_-20 to C-17/C-18/C-19, from H_2_-15 to C-3/C-4, from H_3_-25 to C-22/C-23/C-24, and from H_2_-21 to C-4/C-5, as well as the ^1^H-^1^H COSY cross peaks of H_2_-15/H_2_-16/H-17 and H_2_-5/H-4/H_2_-21/H-22. The fragment (CH_2_-26-CH-27-C-28-CH_3_-29-CH_3_-30) was positioned at C-8, which was deduced from the HMBC cross peaks from H_3_-30 to C-27/C-28/C-29, and from H_2_-26 to C-8/C-9, as well as the ^1^H-^1^H COSY interactions of H_2_-26/H-27 (Figures S4–S6). Furthermore, a methoxy group was located at C-1 due to the HMBC correlation. Thus, the planar construction of compound **1** was finally built (Figure 2).

**Figure 3:**
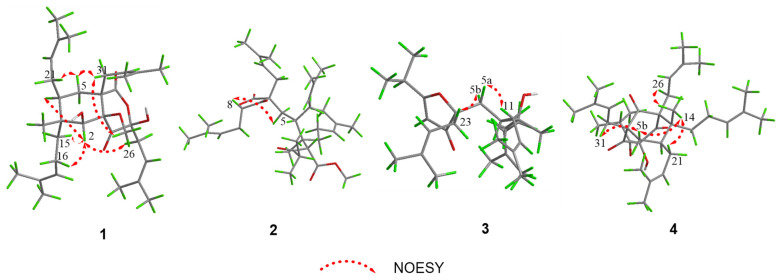
Key NOESY correlations of compounds **1**–**4**.

**Figure 4:**
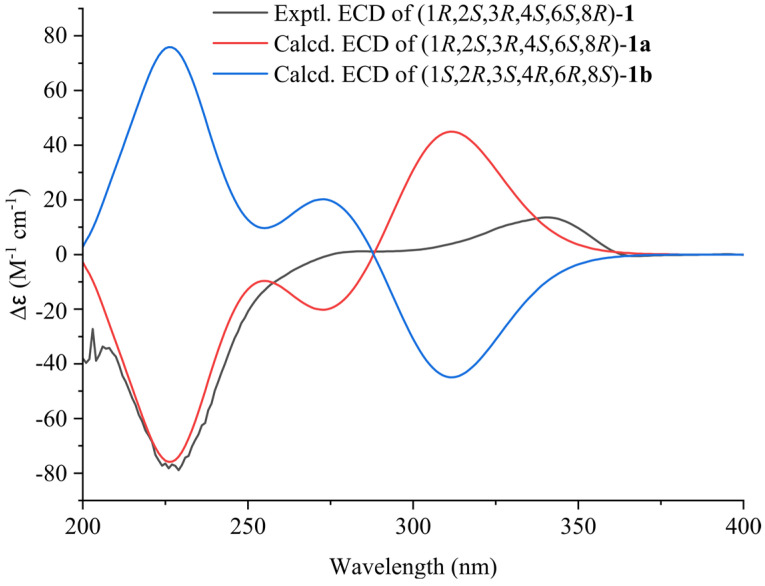
Calculated and experimental ECD spectra of compound **1**.

The relative configuration of compound **1** was elucidated by the NOESY data (Figure 3). The NOESY correlations of H-2/H-15b, H-2/H-4, H-2/H_2_-26b, H-5a/H-21a, H-5a/H-31b, and H-31b/H_3_-OCH_3_ indicated that H-2 and the isoprenyl group at C-3 and C-8 were *α*-oriented; moreover, the isoprenyl group at C-4 and C-6 and the methoxy group were in the same *β-*orientation (Figure S7). From the above analysis, the relative configuration of compound **1** was determined to be 1R*, 2S*, 3R*, 4S*, 6S*, 8R*. Moreover, the absolute configuration of (1*R*, 2*S*, 3*R*, 4*S*, 6*S*, 8*R*)-**1a** was determined using the calculated ECD data, showing good agreement with the experimental data (Figure 4).

Hyperforatum B (**2**) was purified as a colorless oil. The molecular formula of C_36_H_56_O_6_ was confirmed by its HRESIMS data (*m/z*: [M + Na]^+^ calcd. 607.3969; found 607.3958), indicating 9 degrees of unsaturation (Figure S18). The ^1^H NMR data of compound **2** displayed signals for four olefinic protons (*δ*_H_ 5.16, 1H, t, *J* = 7.0 Hz; 5.01, 1H, t, *J* = 7.0 Hz; 4.95, 2H, overlap), nine methyls (*δ*_H_ 1.08–1.72, s), and an isopropyl group (*δ*_H_ 2.66, 1H, m; 1.07, 3H, d, *J* = 6.7 Hz; 1.04, 3H, d, *J* = 6.7 Hz). The ^13^C NMR and DEPT spectra indicated 36 carbons, including 11 methyls, 6 methylenes, 8 methines, 1 methoxyl, and 10 quaternary carbons. The comprehensive analysis of 2D NMR revealed that compound **2** shared the same planar structure as compound **5** [17] (Figure 2 and Figures S11–S16). However, minor deviations were observed: C-4 (*δ*_C_ 41.6), C-5 (*δ*_C_ 37.5), C-21 (*δ*_C_ 30.2) in compound **2** were replaced by C-4 (*δ*_C_ 40.7), C-5 (*δ*_C_ 37.2), C-21 (*δ*_C_ 35.5) in compound **5**, respectively. Moreover, the chemical shifts of H_2_-21 and H-22 also changed significantly (Table 1). These deviations might have resulted from the differences in the configurations of the isoprenyl group at C-4. When the orientation of the isoprenyl group was changed at the C-4 position, the two large groups attached at the ends of the C-4 position had steric hindrance effects, which might have led to a significant difference of chemical shifts around the C-4 position. The deduction cannot be confirmed by the key NOESY correlations because the isoprenyl group was located in a flexible side chain. The NOESY correlations of H-8/H-5a indicated that the stereochemistry of C-8 was *α*-oriented (Figure 3 and Figure S17). The absolute configuration of compound **2** was unable to be calculated due to the chiral center being on a flexible chain.

The molecular formula of hyperforatum C (**3**) was deduced as C_27_H_44_O_3_ based on its HRESIMS data (*m/z*: [M + Na]^+^ calcd 439.3183, found 439.3177), suggesting 6 unsaturation sites (Figure S28). The ^1^H NMR spectrum showed the presence of 3 olefinic protons (*δ*_H_ 5.11, 2H, overlap; 4.93, 1H, t, *J* = 7.2 Hz), 7 singlet methyls (*δ*_H_ 1.03–1.69, s), and an isopropyl (*δ*_H_ 2.69, 1H, m; 1.22, d, 3H, *J* = 6.8 Hz; 1.20, d, 3H, *J* = 6.8 Hz). The ^13^C NMR data of compound **3** displayed 27 carbons assigned to 9 methyls, 5 methylenes, 6 methines (4 olefinic), and 7 nonprotonated carbons (1 carbonyl, 4 olefinic, 2 oxygenated). The above analysis suggested that compound **3** should be a monocyclic PPAP (Figures S21–S23). The HMBC interactions from H_3_-22 to C-19/C-20/C-21 along with the ^1^H-^1^H COSY cross peaks of H_2_-4/H-5/H_2_-18/H-19 constructed the fragment A (C-4-C-5-C-18-C-19-C-20-C-21-C-22). The HMBC interactions from H_3_-17 to C-14/C-15/C-16 along with the ^1^H-^1^H COSY cross peaks of H_2_-12/H_2_-13/H-14 constructed the fragment B (C12-C-13-C-14-C-15-C-16-C-17). The HMBC interactions from H_3_-27 to C-24/C-25/C-26 accompanied by the ^1^H-^1^H COSY cross peaks of H_2_-23/H-24 constructed the fragment C (C-23-C-24-C-25-C-26-C-27). The fragment C was located at C-6 due to the HMBC correlations from H_2_-23 to C-6. The fragments A and B were connected through the carbon C-3, of which the result was supported by the HMBC interactions from H_3_-11 to C-3/C-4/C-12. In addition, the HMBC correlations from H_3_-9 to C-7/C-10/C-11, from H-2 to C-1/C-7, from H_2_-23 to C-1/C-6, and from H_2_-5 to C-6, as well as the ^1^H-^1^H COSY cross peaks of H_3_-9/H-8/H_3_-10, formed the architecture of furanone with a isopropyl at carbon C-7 (Figure 2 and Figures S24–S26). Compound **3** might be obtained with a rearrangement of monocyclic polyprenylated acylphloroglucinols (MPAPs). The relative configuration of compound **3** could not be determined by the NOESY correlations because its chiral center was located in the flexible chain. Meantime, ECD calculations were quite challenging in the determination of the absolute configuration of compound **3**. Unfortunately, its crystal failed to be obtained after the solvent conditions were changed multiple times.

Hyperforatum D (**4**) was isolated as a colorless oil. The molecular formula of C_35_H_52_O_4_ was supported by its HRESIMS data (*m/z* [M + H]^+^ calcd 537.3938, found 537.3928), with 10 degrees of unsaturation (Figure S38). The ^1^H NMR and ^13^C spectra of compound **4** in CDCl_3_ showed a paired mixture of two keto-enol tautomers (**4a** and **4b**) in an approximate 1:1 ratio. The keto–enol tautomerism of a β,β′-triketo moiety was easily converted for the PPAPs seen in hypascyrins A–E [18]. The ^1^H NMR spectrum of compound **4a** revealed the presence of 4 olefinic protons (*δ*_H_ 5.20, 1H, *J* = 7.0 Hz; 5.02, 1H, overlap, 4.84, 1H, t, *J* = 7.2 Hz; 4.76, 1H, *J* = 6.2 Hz), 9 singlet methyls (*δ*_H_ 1.22–1.70, s), an isopropyl group (*δ*_H_ 3.82, 1H, m; 1.20, 3H, d, *J* = 6.8 Hz; 1.06, 3H, d, *J* = 6.8 Hz) (Table 2). The ^13^C NMR spectrum combined with HSQC and HMBC revealed that tautomer **4a** was a type B PPAP derivative with a bicyclo [3.3.1]nonane-2,4,9-trione system, whose structure was similar to spiranthenone B [19]. The main differences were the presence of an isopropyl group at C-8 and a prenyl group at C-3 in tautomer **4a**. The deduction was verified by the HMBC correlations from H_3_-20 to C-17/C-18/C-19, from H_2_-15 to C-2/C-3/C-4/C-17, and from H_3_-12 to C-10/C-11/C-13, along with ^1^H-^1^H COSY correlations of H_2_-15/H_2_-16 and H_3_-12/H-11/H_3_-13 (Figure 2 and Figures S31–S36). For tautomer **4b,** the hydroxyl group was located at the C-7 position, while the carbonyl was located at the C-9 position; these conclusions were confirmed by the HMBC correlations from H_2_-26 (*δ*_H_ 2.65, 2.62) to C-1 (*δ*_C_ 207.8)/C-2 (*δ*_C_ 67.1)/C-3 (*δ*_C_ 50.6)/C-9 (*δ*_C_ 200.7), and from H_2_-31 (*δ*_H_ 2.51, 2.48) to C-1 (*δ*_C_ 207.8)/C-6 (*δ*_C_ 64.2)/C-7 (*δ*_C_ 194.2)/C-5 (*δ*_C_ 37.4). Comprehensive analysis of the 2D NMR data also revealed the 2D structure of the tautomer (**4b**) (Figure 2 and Figures S31–S36). The relative configurations of tautomers **4a** and **4b** were determined using the NOESY data (Figure 3). The NOESY correlations of H_3_-14/H_2_-21, H_3_-14/H-5b, and H-5b/H_2_-30 indicated that these protons were in the same *β* orientation (Figure S38). Its relative configuration was equal to that of spiranthenone B based on the NOESY cross peaks. Finally, the relative configuration of compound **4** was confirmed, as shown in Figure 3.

When comparing the spectroscopic data to those reported in the literature, thirteen known compounds were identified to be methyl (α*S*,β*R*,γ*S*,3*S*)-tetrahydro-β-methyl-γ,3,5-tris(3-methyl-2-buten-1-yl)-α-(2-methyl-1-oxopropyl)-β-(4-methyl-3-penten-1-yl)-2,4-dioxo-3-furanpentanoate (**5**) [17], lupeol acetate (**6**) [20], lup -20(29)-en-3-one (**7**) [21], α–amyrin acetate (**8**) [22], methyl oleanolate (**9**) [23], (–)-(6aR,11aR)-homopterocarpin (**10**) [24], 5-formyl-7,8-dimethyltocol (**11**) [25], cassipourol (**12**) [26], ent-α-cyperone (**13**) [27], mullein (**14**) [28], ledol (**15**) [29], kobusone (**16**) [30], and dihydroactinidiolide (**17**) [31].

**Figure 5:**
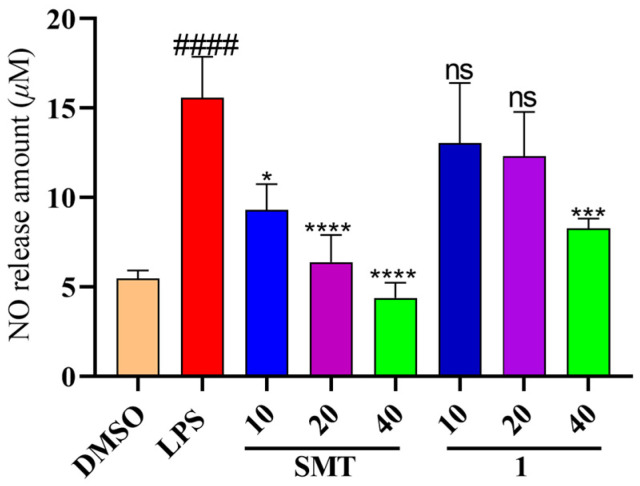
Inhibition effect of compound **1** on the production of NO in BV-2 cells treated with LPS. DMSO (blank control); LPS (model group); SMT (positive control); **1** (compound **1**). Results are expressed as mean ± SEM, ns = no significant difference, * *p* < 0.05, *** *p* < 0.001, and **** *p* < 0.0001, ^####^ *p* < 0.0001, compared to the LPS (one-way ANOVA).

There are many reports on the anti-Alzheimer’s effects of *H. perforatum* [14,32]. Chronic inflammation is an important cause of the development of Alzheimer’s disease’s pathogenesis [33]. The production of nitric oxide (NO) in LPS-stimulated microglial cells is used as a cellular model to evaluate the effects of anti-neuroinflammation. Since we ended up with insufficient quantities of compounds **2**, **3**, and **4** to complete activity evaluation, we assessed the biological activity of compound **1** only. Consequently, compound **1** significantly inhibited NO production at 40 μM (Figure 5). However, its anti-inflammatory mechanisms need to be further explored.

## 3. Materials and Methods

### 3.1. General

NMR spectra were carried out on a Bruker Avance Neo at 400 MHz and 800 MHz (Bruker BioSpin, Fällanden, Switzerland) using tetramethylsilane (TMS) as internal standard. Optical rotations (ORs) were recorded on an Autopol III automatic polarimeter (Rudolph Research Analytical). A Chirascan spectrometer was used to obtain the UV and experimental CD spectra. HRESIMS data were obtained on a LC-30A + TripleTOF5600+ (AB Sciex Pte. Ltd., Framingham, MA, USA). Separations and purifications of the samples were conducted on silica gel (200–300 and 300–400 mesh, Qingdao Marine Chemical Ltd., Qingdao, China), ODS RP-C_18_ (50 μm, YMC Co., Ltd., Kyoto, Japan), and Sephadex LH-20 (40–70 μm, Amersham Pharmacia Biotech AB, Stockholm, Sweden). A shimadzu LC-20AP liquid chromatography system equipped with a reversed-phase (RP) C-18 column (10 mm × 250 mm, 5 μm) was applied to complete sample purification.

### 3.2. Plant Materials

Air-dried aerial portions of *H. perforatum* were collected in August 2018 from Shangluo City, China. The plant was identified by Pro. Zhen-hai Wu. The sample (no. 20180805HPL) was preserved at Shaanxi Key Laboratory of Natural Products and Chemical Biology, Northwest A&F University.

### 3.3. Extraction and Isolation

Air-dried aerial portions of *H. perforatum* (100 kg) were powdered and extracted using 95% EtOH (300 L × 3, each of 2 h) via three cycles of refluxing. The filtered solution was then concentrated under reduced pressure to obtain the crude extract, which was suspended in water and then partitioned with n-hexane and EtOAc. The n-hexane fraction (1.03 kg) was subjected to silica gel column elution with petroleum ether/EtOAc (100:0 to 1:1, *v/v*) to obtain six fractions (Fr. 1–6). Fr.3 (291.0 g) was applied to a silica gel column and eluted with petroleum ether/EtOAc (100:1 to 20:1, *v/v*) to yield six subfractions (Fr. 3A–3F). Fr.3B (27.2 g) was further fractionated using a RP-C18 CC (MeOH/H_2_O, 90:10 to 100:0, *v*/*v*) and a silica gel CC (PE/EtOAc, 100:0 to 30:1) to obtain four subfractions (Fr.3Ba–3Bd). Fr.3Bb (93.3 mg) was purified via preparative HPLC using MeOH/H_2_O (91:9, *v*/*v*, 2 mL/min) isocratic elution to yield compounds **6** (6.0 mg, t_R_ = 50 min), **3** (2.2 mg, 55 min) and **2** (6 mg, t_R_ = 60 min). Fr.3Bd (85.0 mg) was subjected to preparative HPLC using CH_3_CN:H_2_O (93:7, *v*/*v*, 2 mL/min) isocratic elution to yield compounds **1** (4.9 mg, t_R_ = 46 min) and **4** (12.7 mg, t_R_ = 75 min); the column temperature for HPLC was 37 °C. Fr.3E (131.7 g) was subjected to a RP-C18 CC eluted with MeOH:H_2_O (60:40 to 100:0, *v/v*) to obtain four subfractions (Fr.3Ea–3Ed). Fr.3Ea (187.0 mg) was loaded onto a Sephadex LH-20 column using CH_2_Cl_2_:MeOH (1:1, *v/v*) and then purified via semipreparative HPLC (CH_3_CN:H_2_O, 46:54, *v*/*v*, 2 mL/min) to afford compounds **16** (15.0 mg, t_R_ = 27 min) and **17** (6.3 mg, t_R_ = 36 min). Fr.3Ec (3.96 g) was fractionated using a silica gel column and eluted with n-hexane:EtOAc (50:1 to 1:1, *v*/*v*) to obtain five subfractions (Fr.3Ec1–3Ec5). Fr.3Ec2 (446.0 mg) was repurified by semi-preparative HPLC (MeOH:H_2_O, 46:54, *v*/*v*, 2 mL/min) to acquire compounds **13** (2.7 mg, t_R_ = 22 min), **14** (2.8 mg, t_R_ = 26 min), and **15** (11.0 mg, t_R_ = 38 min). Fr.3Ec4 (2.11 g) was fractionated by a Sephadex LH-20 column using CH_2_Cl_2_:MeOH (1:1, *v/v*) and further separated by a silica gel column and eluted with petroleum ether/EtOAc (50:1 to 1:1, *v/v*) to obtain compounds **6** (187.6 mg), **7** (79.1 mg), **8** (11.0 mg), **9** (47.6 mg), **10** (7.0 mg), **11** (8.5 mg), and **12** (7.5 mg).

### 3.4. Structural Elucidation

Hyperforatum A (**1**), colorless oil; [*α*]^20^_D_ +2 (*c* 0.1 MeOH); UV (MeOH) λ_max_ (log *ε*) 200 (4.25), nm; ECD (MeOH) λ_max_ (∆ε) 226 (−25.16), 340 (4.36) nm; ^1^H and ^13^C NMR data (see Table 1); HRESIMS *m/z* 513.3573 [M – H_2_O + H]^+^ (calcd. for C_32_H_49_O_5_, 513.3574).

Hyperforatum B (**2**), colorless oil; [*α*]^20^_D_ +7.5 (*c* 1.00 MeOH); UV (MeOH) λ_max_ (log *ε*) 200 (3.71) nm; ECD (MeOH) λ_max_ (∆*ε*) 237 (−3.36) nm; ^1^H and ^13^C NMR data (see Table 1); HRESIMS *m/z* 607.3958 [M + Na]^+^ (calcd. for C_36_H_56_O_6_Na, 607.3969).

Hyperforatum C (**3**), colorless oil; [*α*]^20^_D_ +5.9 (*c* 0.35 MeOH); UV (MeOH) λ_max_ (log *ε*) 200 (4.20), 262 (3.34) nm; ECD (MeOH) λ_max_ (∆ε) 231 (−0.97), 302 (0.82) nm; ^1^H and ^13^C NMR data (see Table 2); HRESIMS *m/z* 439.3177 [M + Na]^+^ (calcd. for C_27_H_44_O_3_Na, 439.3183).

Hyperforatum D (**4**), colorless oil; [*α*]^20^_D_ +4.0 (*c* 1.00 MeOH); UV (MeOH) λ_max_ (log *ε*) 200 (2.85), 244 (2.14), 289 (2.19) nm; ECD (MeOH) λ_max_ (∆*ε*) 210 (−1.03), 234 (+0.17), 252 (−0.36), 303 (+0.22) nm; ^1^H and ^13^C NMR data (see Table 2); HRESIMS *m/z* 537.3928 [M + H]^+^ (calcd. for C_35_H_53_O_4_, 537.3938).

### 3.5. Cell Culture

Microglial BV-2 cells from China Center for Type Culture Collection (Wuhan, China) were cultivated in DMEM (Gibco, New York, NY, USA) containing 10% FBS (Gibco) and antibiotics (100 U/mL streptomyces and penicillin) in humidified incubators under 5% CO_2_ at 37 °C.

### 3.6. Measurement of Nitric Oxide (NO) Production

BV-2 cells were seeded in 96-well plates (2 × 10^5^ cells/mL) overnight. The cells were treated with LPS (2 μg/mL) and various concentrations of hyperforatum A (**1**) (10, 20, 40 μM) for 24 h, with S-Methylisothiourea (SMT) as the positive control. The production of NO was measured in cell supernatants with a Griess reagent. The absorbance was recorded at 540 nm using a microplate reader. The MTT method was applied to determine the cell viability after incubation using the test compound.

### 3.7. Statistical Analysis

All data were presented as mean ± SD and analyzed with GraphPad Prism 9.0 software. The significant differences between different groups were performed using one-way ANOVA multiple comparisons.

## 4. Conclusions

The phytochemical components of the PPAPs and terpenoids were investigated from the aerial parts of *H. perforatum*. Seventeen secondary metabolites, including five PPAPs and nine terpenoids, were isolated and identified from the title plants. This study reported two unusual carbon cores of PPAPs, of which hyperforatum A was a chromene-1,4-dione bicyclic system, and hyperforatum B and C possessed rare monocyclic features. The new compound, hyperforatum A (**1**), displayed a moderate inhibitory capacity on LPS-induced NO production.

## Figures and Tables

**Table 1:** ^13^C NMR and ^1^H NMR data of compounds **1**, **2**, and **5**.

No	1 *^a^*		2 *^a^*		5 *^a^*	
	*δ*_C_	*δ*_H_ (*J* in Hz)	*δ*_C_	*δ*_H_ (*J* in Hz)	*δ*_C_	*δ*_H_ (*J* in Hz)
1	80.9		169.8		169.8	
2	66.6	3.39 s	61.4	3.91 s	61.5	4.02 s
3	37.3		44.2		44.1	
4	41.7		41.6	1.82 m	40.7	1.88 m
5	33.9	1.84 d (7.5)	37.5	1.98 m	37.2	2.00 m
				1.69 m		1.68 m
6	47.7		54.0		53.9	
7	180.3		176.2		175.9	
8	97.7		84.6	4.53 dd (8.7, 4.6)	84.7	4.35 dd (8.5, 4.4)
9	205.1		212.6		212.6	
10			208.6		208.6	
11			42.8	2.66 m	42.9	2.66 m
12			18.3	1.04 d (6.7)	18.3	1.04 d (6.8)
13			18.6	1.07 d (6.7)	18.7	1.07 d (6.8)
14	18.3	0.91 s	21.2	1.05 s	21.3	1.08 s
15	42.1	1.55 m	35.6	2.39 m	35.2	1.56 m
		1.26 m		1.53 m		
16	22.6	2.01 m	22.9	1.93 m	23.0	1.96 m
				1.82 m		1.84 m
17	123.9	5.05 m	124.7	5.02 t (7.0)	124.8	5.01 t (7.0)
18	132.3		131.5		131.4	
19	18.1	1.61 s	18.0	1.61 s	17.9	1.61 s
20	26.0	1.68 s	25.9	1.65 s	25.9	1.66 s
21	28.0	2.14 m	30.2	2.03 m	35.6	2.48 m
		1.59 s		1.82 m		2.35 m
22	122.8	5.05 m	124.4	4.95 t (7.0)	124.4	5.08 t (7.1)
23	133.2		132.7		131.6	
24	18.1	1.57 s	18.2	1.55 s	18.2	1.61 s
25	25.8	1.69 s	25.9	1.65 s	26.0	1.72 s
26	26.1	2.58 dd (15.5, 7.0)	29.6	2.46 m	30.3	2.58 m
		2.50 dd (15.5, 7.6)		2.34 m		2.42 m
27	116.1	5.24 m	117.5	5.17 t (7.0)	117.4	5.18 t (7.4)
28	136.2		136.4		136.6	
29	18.0	1.62 s	18.1	1.59 s	18.1	1.63 s
30	26.1	1.71 s	25.9	1.72 s	26.0	1.67 s
31	35.5	2.42 m	35.9	2.46 m	35.2	2.48 m
		2.37 m		2.39 m		2.39 m
32	119.1	5.24 m	117.0	4.97 t (7.0)	116.8	4.96 t (8.0)
33	135.8		137.8		137.4	
34	17.9	1.62 s	17.9	1.61 s	18.0	1.58 s
35	26.3	1.71 s	26.1	1.67 s	26.1	1.67 s
OCH_3_	54.5	3.43 s	52.2	3.69 s	52.2	3.69 s

*^a^* NMR data were recorded in CDCl_3_ (^1^H NMR 400 MHz, ^13^C NMR 100 MHz).

**Table 2:** ^13^C NMR and ^1^H NMR data of compound **3** and tautomers **4a** and **4b**.

No	3 *^a^*		4a *^b^*		4b *^b^*	
	*δ*_C_	*δ*_H_ (*J* in Hz)	*δ*_C_	*δ*_H_ (*J* in Hz)	*δ*_C_	*δ*_H_ (*J* in Hz)
1	207.6		208.1		207.8	
2	102.4	5.42 s	69.9		67.1	
3	74.8		51.2		50.6	
4	43.2	1.49 m	40.3	1.76 m	39.5	1.76, m
5	35.2	2.08 m	39.2	2.11 dd (14.5, 7.3)	37.4	2.02 m
		1.66 m		2.04 m		2.00 m
6	94.3		59.7		64.2	
7	197.9		200.1		194.2	
8	30.6	2.69 m	115.2		114.6	
9	19.6	1.22 d (6.8)	195.4		200.7	
10	20.0	1.20 d (6.8)	207.2		208.2	
11	23.5	1.03 s	35.0	3.82 m	35.6	3.95 m
12	40.4	1.44 m	18.9	1.06 d (6.8)	18.4	1.15 d (6.8)
13	22.1	2.03 m	19.0	1.20 d (6.8)	19.5	1.10 d (6.8)
14	124.9	5.10 t (7.5)	18.8	1.21 s	18.9	1.21 s
15	131.6		36.5	1.38 m	36.1	1.36 m
				1.35 m		1.12 m
16	18.1	1.60 s	29.4	1.84 m	29.1	2.04 m
				1.75 m		1.98 m
17	26.0	1.67 s	124.4	4.84 t (7.2)	124.1	4.87 t (6.2)
18	31.5	2.12 m	133.0		132.9	
		1.95 m				
19	123.9	5.13 t (7.5)	17.8	1.42 s	18.0	1.46 s
20	132.3		26.0	1.66 s	26.1	1.66 s
21	18.1	1.70 s	22.9	1.88 m	22.6	1.80 m
				1.86 m		
22	26.1	1.61 s	123.9	5.02 overlap	123.8	5.02 overlap
23	35.7	2.52 dd (14.6, 8.4)	132.2		132.1	
		2.34 dd (14.6, 8.4)				
24	116.7	4.93 t (7.5)	17.7	1.57 s	17.7	1.56 s
25	136.0		25.8	1.66 s	25.8	1.66 s
26	17.8	1.64 s	26.2	2.64 m	26.1	2.65 m
				2.52 m		2.62 m
27	25.9	1.59 s	119.8	4.76 t (6.2)	119.1	4.66 t (6.2)
28			134.6		134.4	
29			18.3	1.67 s	18.2	1.65 s
30			26.1	1.67 s	26.0	1.66 s
31			29.9	2.58 m	30.0	2.51 m
				2.51 m		2.48 m
32			119.6	5.20 t (7.0)	120.1	5.15 t (7.2)
33			134.8		134.6	
34			18.2	1.65 s	18.3	1.66 s
35			26.0	1.70 s	26.1	1.67 s

*^a^* NMR data were recorded in CDCl_3_ (^1^H NMR 400 MHz, ^13^C NMR 100 MHz). *^b^* NMR data were recorded in CDCl_3_ (^1^H NMR 800 MHz, ^13^C NMR 200 MHz).

## Data Availability

Supporting data include HRESIMS, UV, CD, and 1D and 2D NMR spectra.

## Supplementary Materials

The following supporting information can be downloaded at: https://www.mdpi.com/article/10.3390/molecules29081756/s1, Figures S1–S7: The 1D and 2D NMR spectra of compound **1** in CDCl_3_, Figure S8: The HRESIMS spectrum of compound **1**, Figures S9 and S10: The UV and ECD spectra of compound **1**, Figures S11–S17: The 1D and 2D NMR spectra of compound **2** in CDCl_3_, Figure S18: The HRESIMS spectrum of compound **2**, Figures S19 and S20: The UV and ECD spectra of compound **2**, Figure S21–S27: The 1D and 2D NMR spectra of compound **3** in CDCl_3_, Figure S28: The HRESIMS spectrum of compound **3**, Figures S29 and S30: The UV and ECD spectra of compound **3**, Figures S31–S37: The 1D and 2D NMR spectra of compound **4** in CDCl_3_, Figure S38: The HRESIMS spectrum of compound **4**, Figures S39 and S40: The UV and ECD spectra of compound **4**.
