# Supplementary material for: Four New Polyprenylated Acylphloroglucinols from Hypericum perforatum L
Source: Molecules. 2024 Apr 12;29(8):1756. doi: 10.3390/molecules29081756 (PMC11052217; doi:10.3390/molecules29081756)

Five new Polycyclic Polyprenylated Acylphloroglucinols from *Hypericum perforatum* L.

Xiaoying Wang[1]†, Wuyang Liu[1]†, Sheng Chen[1], Junmian Tian[1,*], Jinming Gao[1,*]

[1] Shaanxi Key Laboratory of Natural Products & Chemical Biology, College of Chemistry & Pharmacy, Northwest A&F University, Yangling 712100, Shaanxi, People's Republic of China

*Corresponding authors:

Junmian Tian − Tel: +86-29-87092335; Fax: +86-29-87092226; Email: tianjunmian@nwsuaf.edu.cn.

Jinming Gao − Tel: +86-29-87092335; Fax: +86-29-87092226; Email: jinminggao@nwsuaf.edu.cn.

Contents

Figure S1. The ^{1}H NMR spectrum of Hyperforatum A (1) in $CDCl_3$ (400 MHz)....................3
Figure S2. The ^{13}C NMR spectrum of Hyperforatum A (1) in $CDCl_3$ (100 MHz)...................3
Figure S3. The DEPT 135° spectrum of Hyperforatum A (1) in $CDCl_3$...................................4
Figure S4. The ^{1}H-^{1}H COSY spectrum of Hyperforatum A (1)...4
Figure S5. The HSQC spectrum of Hyperforatum A (1)...5
Figure S6. The HMBC spectrum of Hyperforatum A (1)...5
Figure S7. The NOESY spectrum of Hyperforatum A (1). 6
Figure S8. The HRESIMS spectrum of Hyperforatum A (1). 6
Figure S9. The UV spectrum of Hyperforatum A (1). 7
Figure S10. The Experimental ECD spectrum of Hyperforatum A (1). 7
Figure S11. The ^{1}H NMR spectrum of Hyperforatum B (2) in $CDCl_3$ (400 MHz). 8
Figure S12. The ^{13}C NMR spectrum of Hyperforatum B (2) in $CDCl_3$ (100 MHz). 8
Figure S13. The DEPT 135° spectrum of Hyperforatum B (2) in $CDCl_3$. 9
Figure S14. The ^{1}H-^{1}H COSY spectrum of Hyperforatum B (2). 9
Figure S15. The HSQC spectrum of Hyperforatum B (2). 10
Figure S16. The HMBC spectrum of Hyperforatum B (2). 10
Figure S17. The NOESY spectrum of Hyperforatum B (2). 11
Figure S18. The HRESIMS spectrum of Hyperforatum B (2). 11
Figure S19. The UV spectrum of Hyperforatum B (2)..12
Figure S20. The Experimental ECD spectrum of Hyperforatum B (2)..12
Figure S21. The ^{1}H NMR spectrum of Hyperforatum C (3) in $CDCl_3$ (400 MHz)..............13
Figure S22. The ^{13}C NMR spectrum of Hyperforatum C (3) in $CDCl_3$ (100 MHz). 13
Figure S23. The DEPT 135° spectrum of Hyperforatum C (3) in $CDCl_3$.................................14
Figure S24. The ^{1}H-^{1}H COSY spectrum of Hyperforatum C (3). 14
Figure S25. The HSQC spectrum of Hyperforatum C (3). 15
Figure S26. The HMBC spectrum of Hyperforatum C (3). 15
Figure S27. The NOESY spectrum of Hyperforatum C (3)...16
Figure S28. The HRESIMS spectrum of Hyperforatum C (3)...16
Figure S29. The UV spectrum of Hyperforatum C (3). 17
Figure S30. The Experimental ECD spectrum of Hyperforatum C (3). 17
Figure S31. The ^{1}H NMR spectrum of Hyperforatum D (4) in $CDCl_3$ (800 MHz). 18
Figure S32. The ^{13}C NMR spectrum of Hyperforatum D (4) in $CDCl_3$ (200 MHz)...............18
Figure S33. The DEPT 135° spectrum of Hyperforatum D (4) in $CDCl_3$. 19
Figure S34. The ^{1}H-^{1}H COSY spectrum of Hyperforatum D (4)...19
Figure S35. The HSQC spectrum of Hyperforatum D (4)..20
Figure S36. The HMBC spectrum of Hyperforatum D (4)..20
Figure S37. The NOESY spectrum of Hyperforatum D (4). 21
Figure S38. The HRESIMS spectrum of Hyperforatum D (4). 21
Figure S39. The UV spectrum of Hyperforatum D (4). 22
Figure S40. The Experimental ECD spectrum of Hyperforatum D (4). 22

Figure S1. The ¹H NMR spectrum of Hyperforatum A (**1**) in $CDCl_3$ (400 MHz).

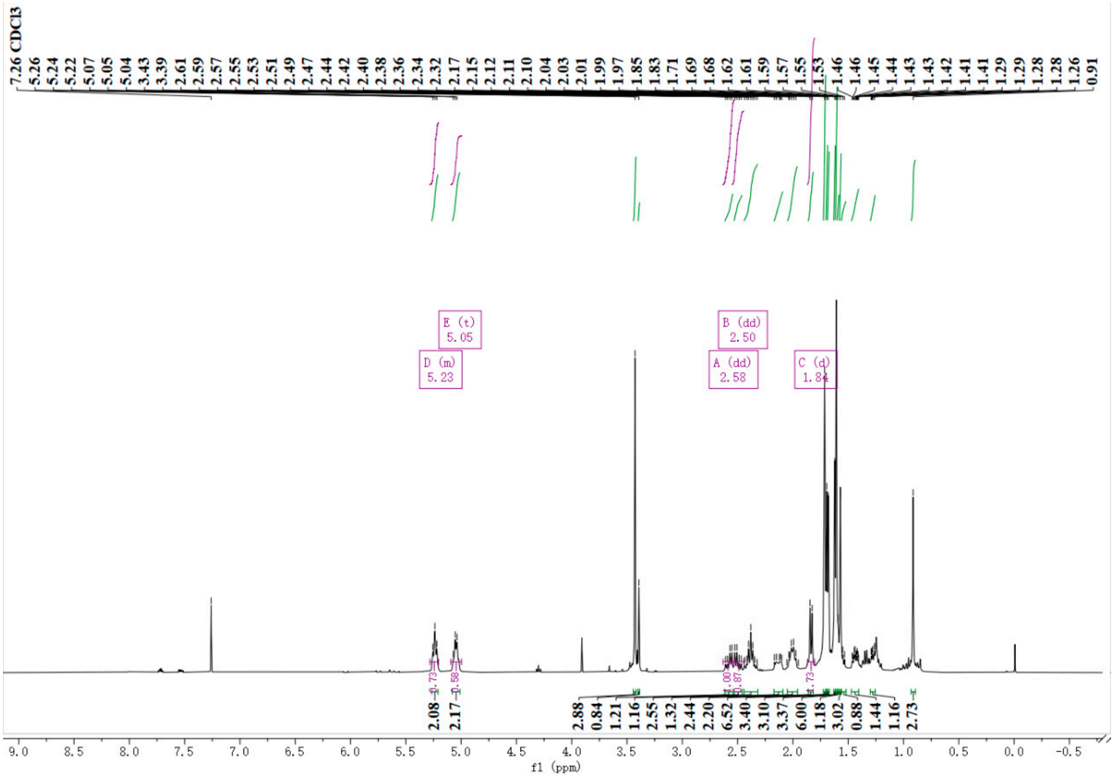

Figure S2. The ¹³C NMR spectrum of Hyperforatum A (**1**) in $CDCl_3$ (100 MHz).

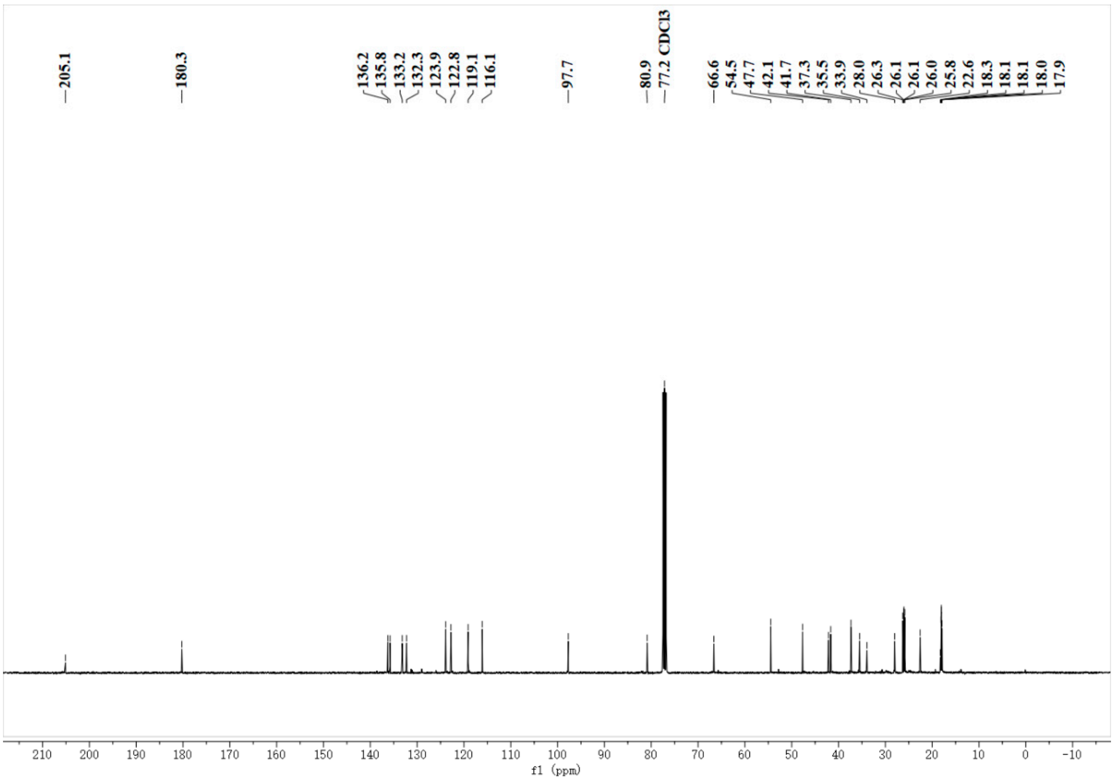

Figure S3. The DEPT 135° spectrum of Hyperforatum A (**1**) in $CDCl_3$.

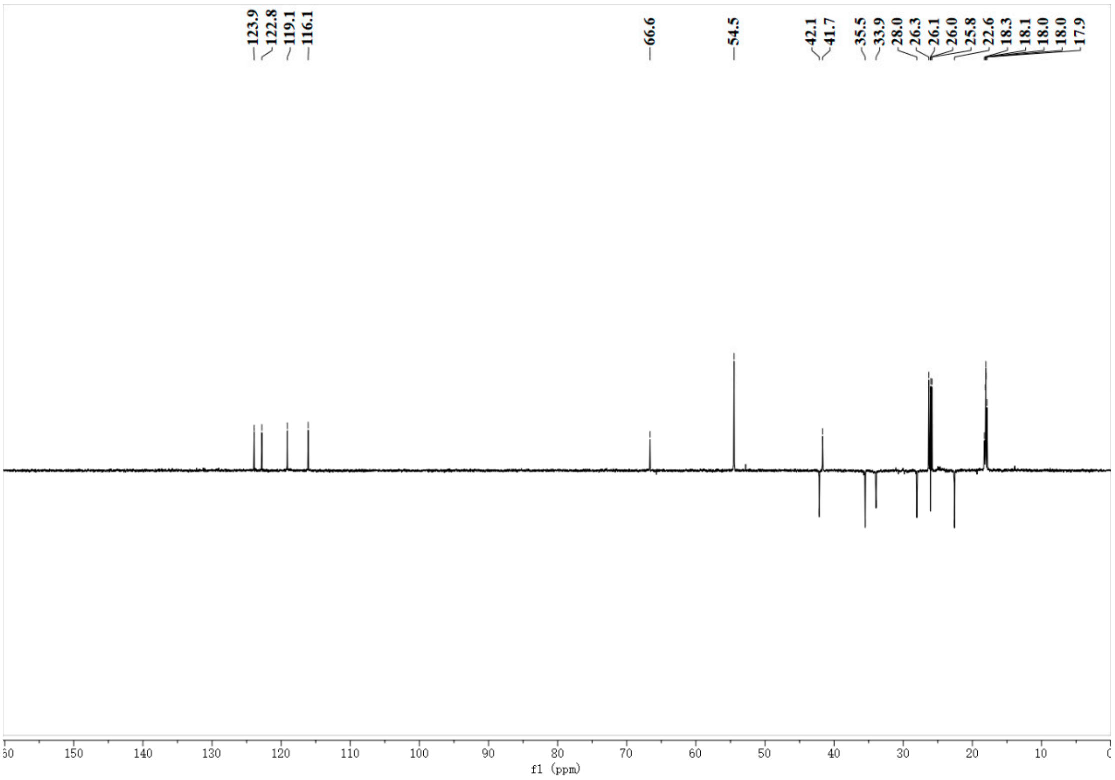

Figure S4. The ^{1}H-^{1}H COSY spectrum of Hyperforatum A (**1**).

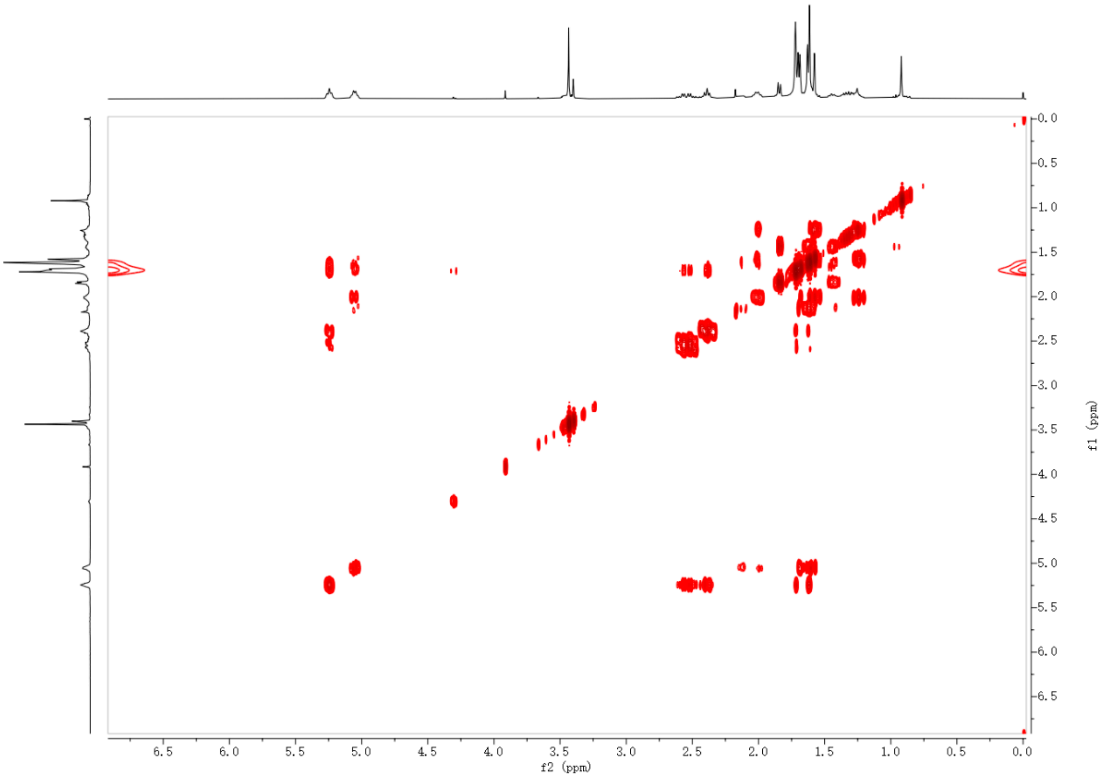

Figure S5. The HSQC spectrum of Hyperforatum A (**1**).

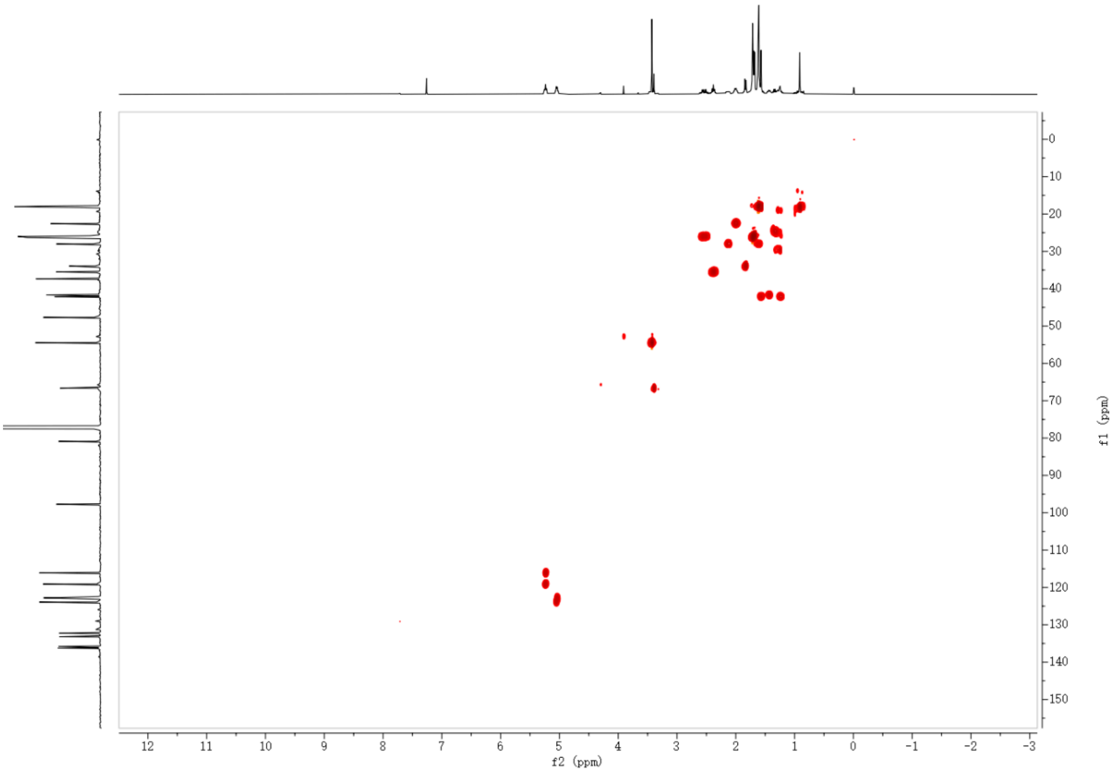

Figure S6. The HMBC spectrum of Hyperforatum A (**1**).

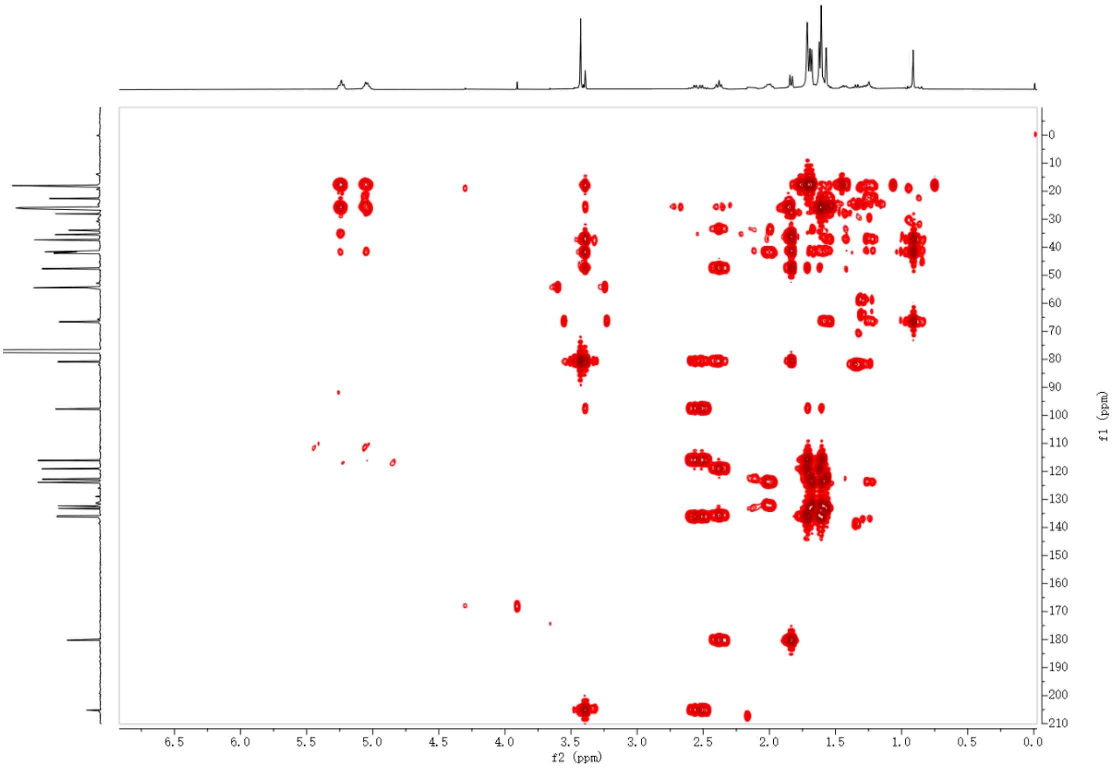

Figure S7. The NOESY spectrum of Hyperforatum A (**1**).

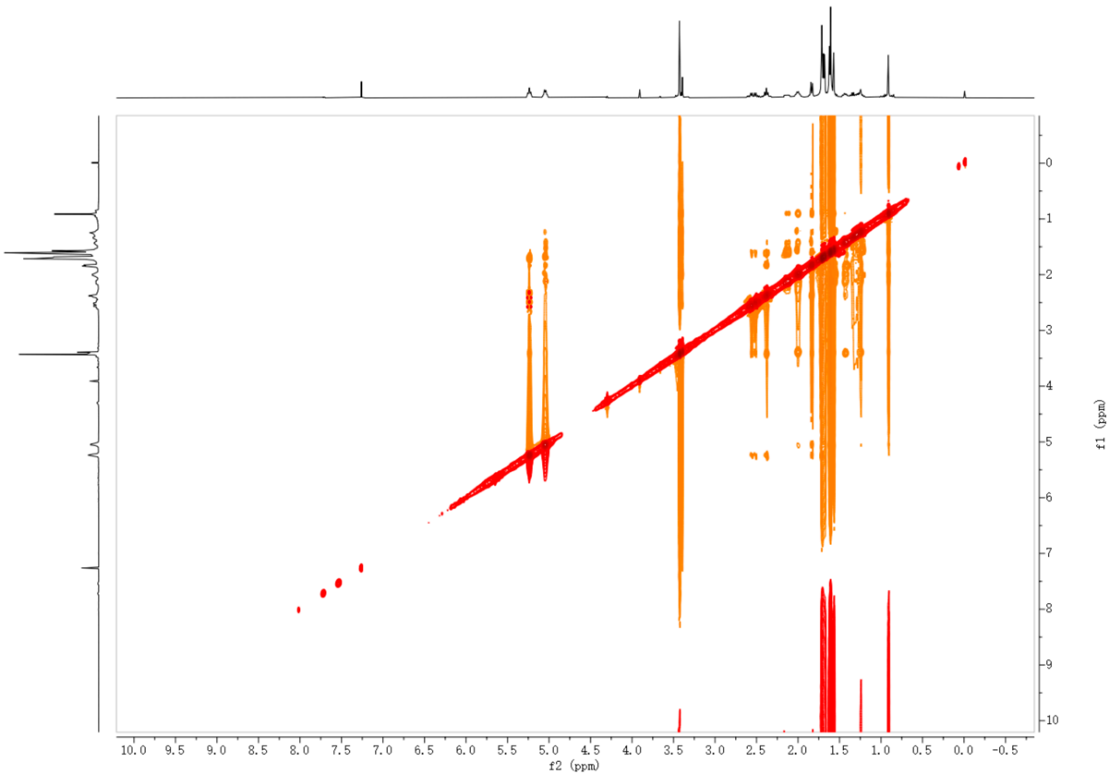

Figure S8. The HRESIMS spectrum of Hyperforatum A (**1**).

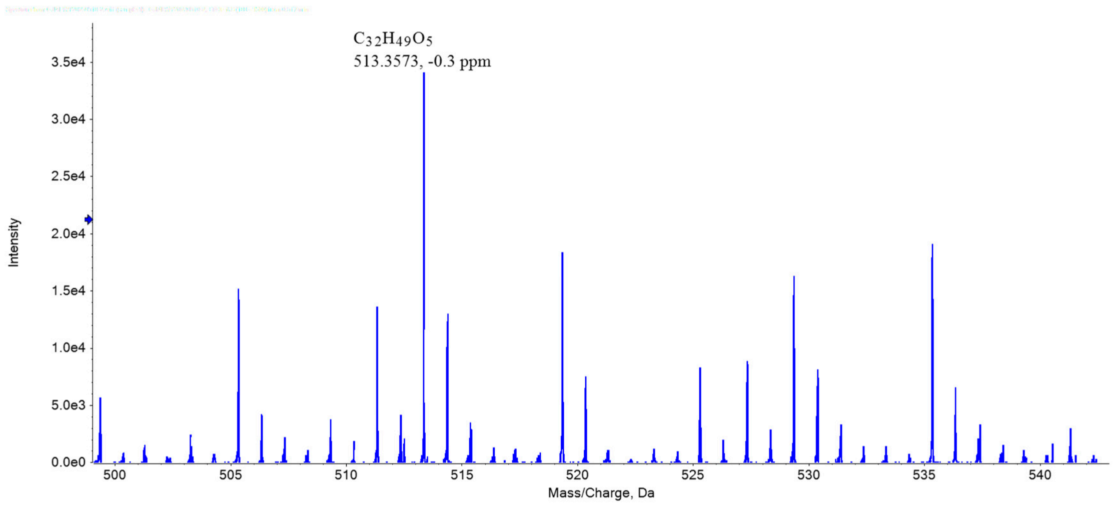

Figure S9. The UV spectrum of Hyperforatum A (**1**).

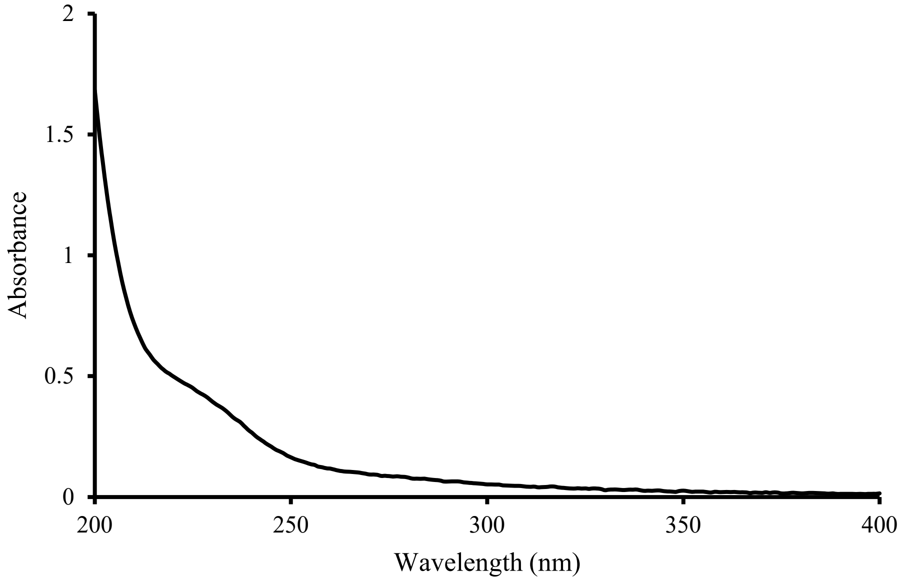

Figure S10. The Experimental ECD spectrum of Hyperforatum A (**1**).

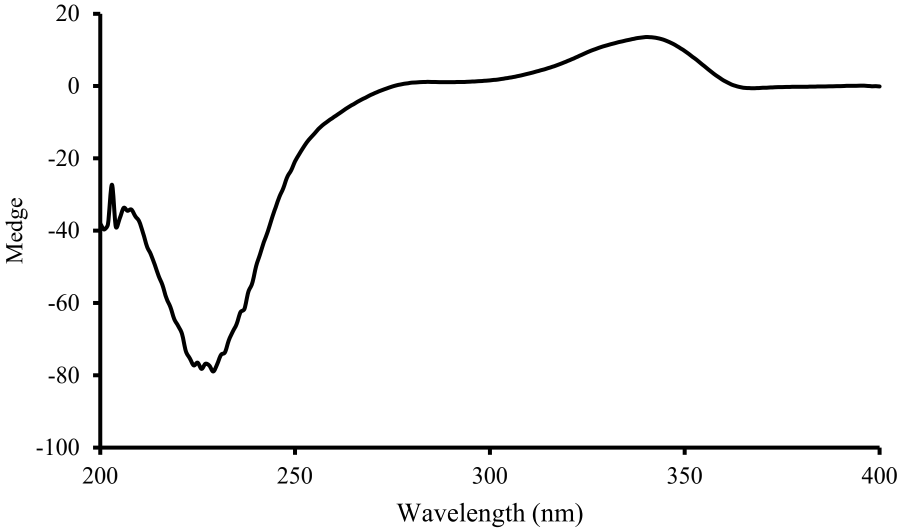

Figure S11. The ^{1}H NMR spectrum of Hyperforatum B (**2**) in $CDCl_3$ (400 MHz).

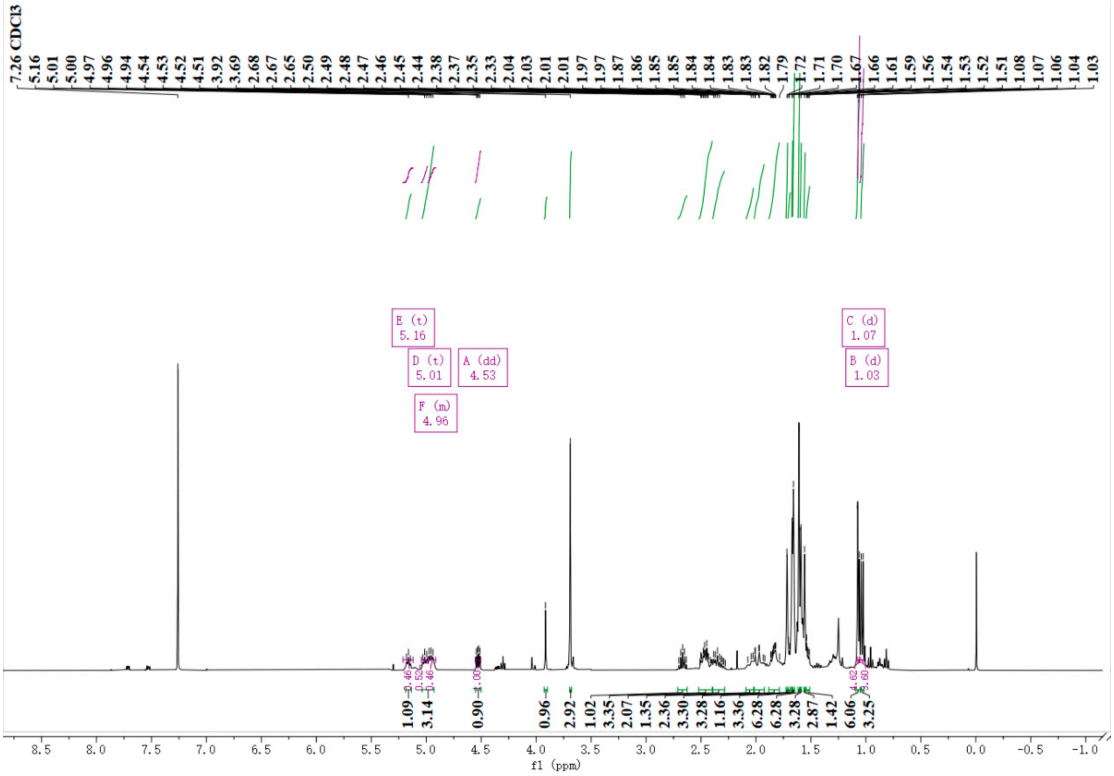

Figure S12. The ^{13}C NMR spectrum of Hyperforatum B (**2**) in $CDCl_3$ (100 MHz).

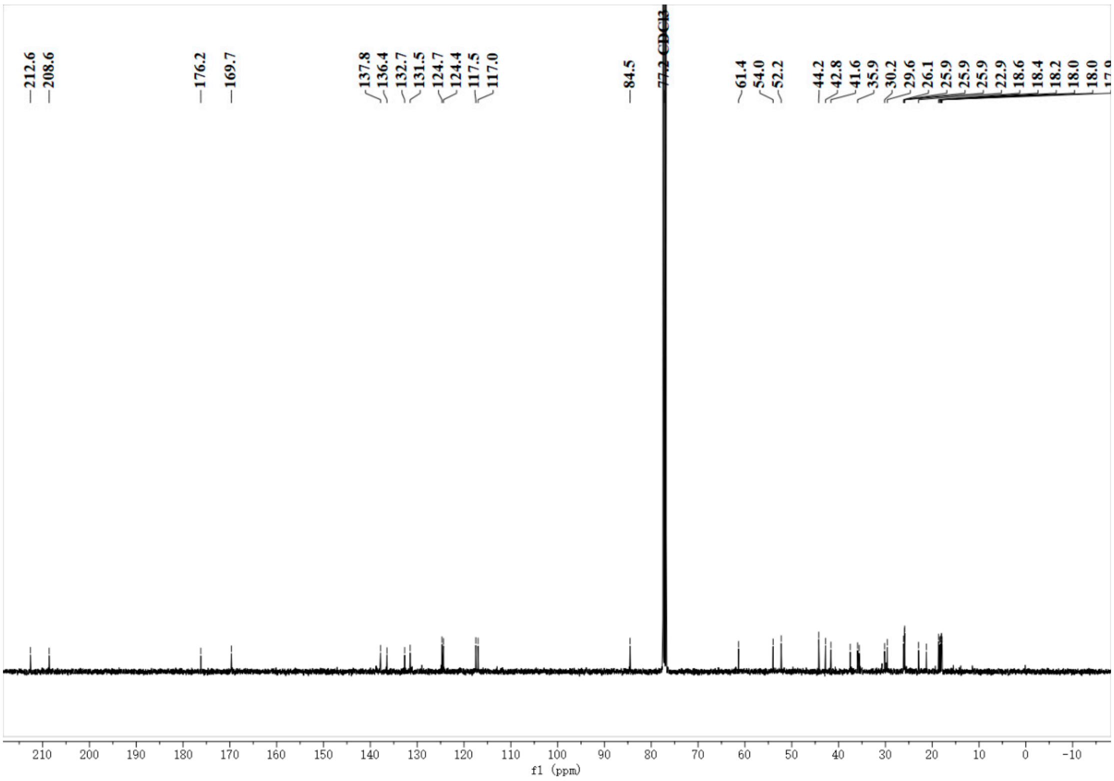

Figure S13. The DEPT 135° spectrum of Hyperforatum B (**2**) in $CDCl_3$.

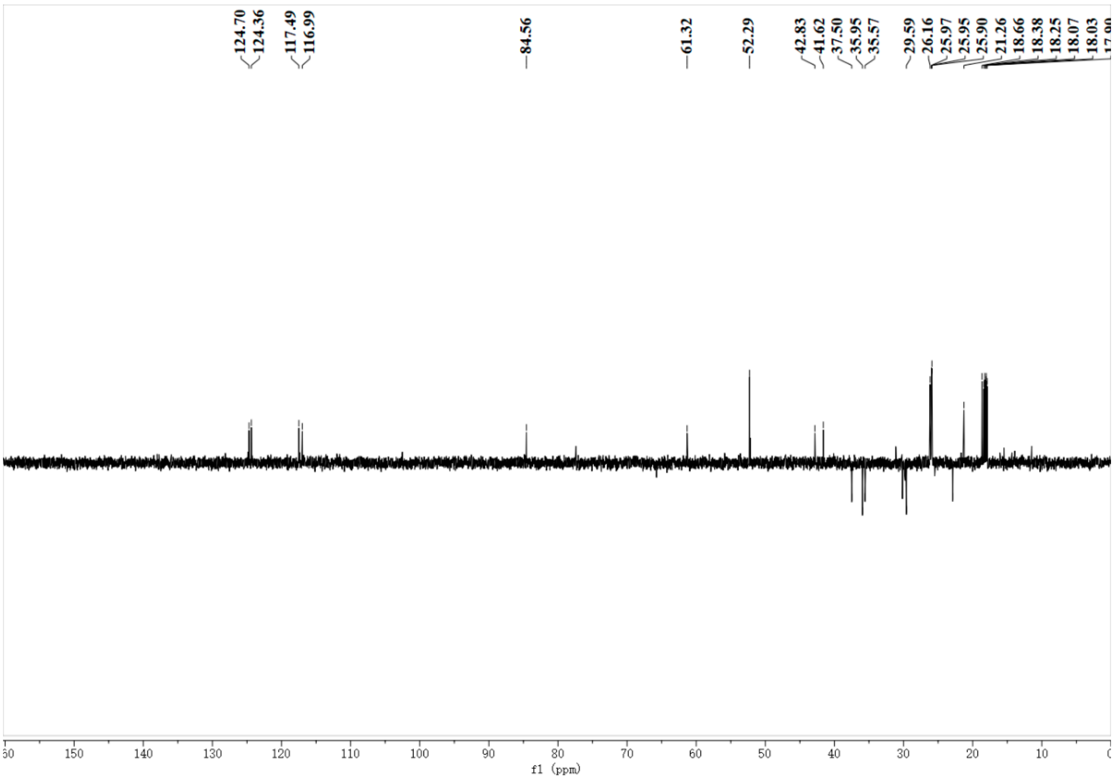

Figure S14. The ^{1}H-^{1}H COSY spectrum of Hyperforatum B (**2**).

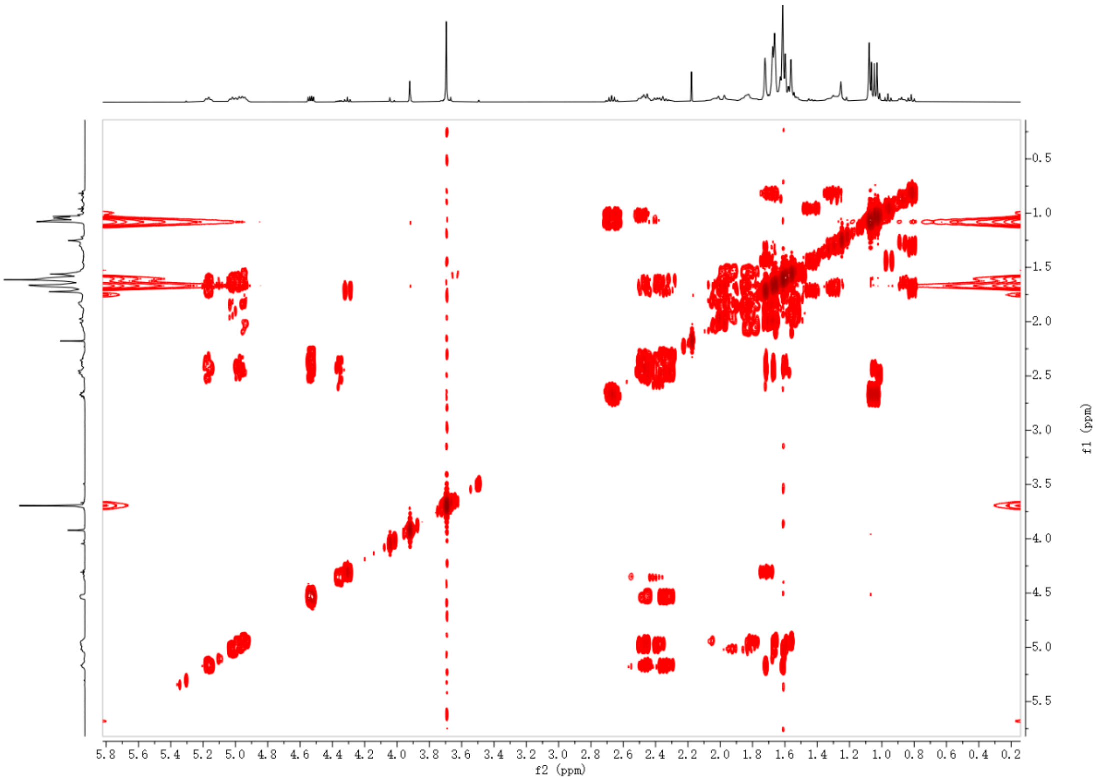

Figure S15. The HSQC spectrum of Hyperforatum B (**2**).

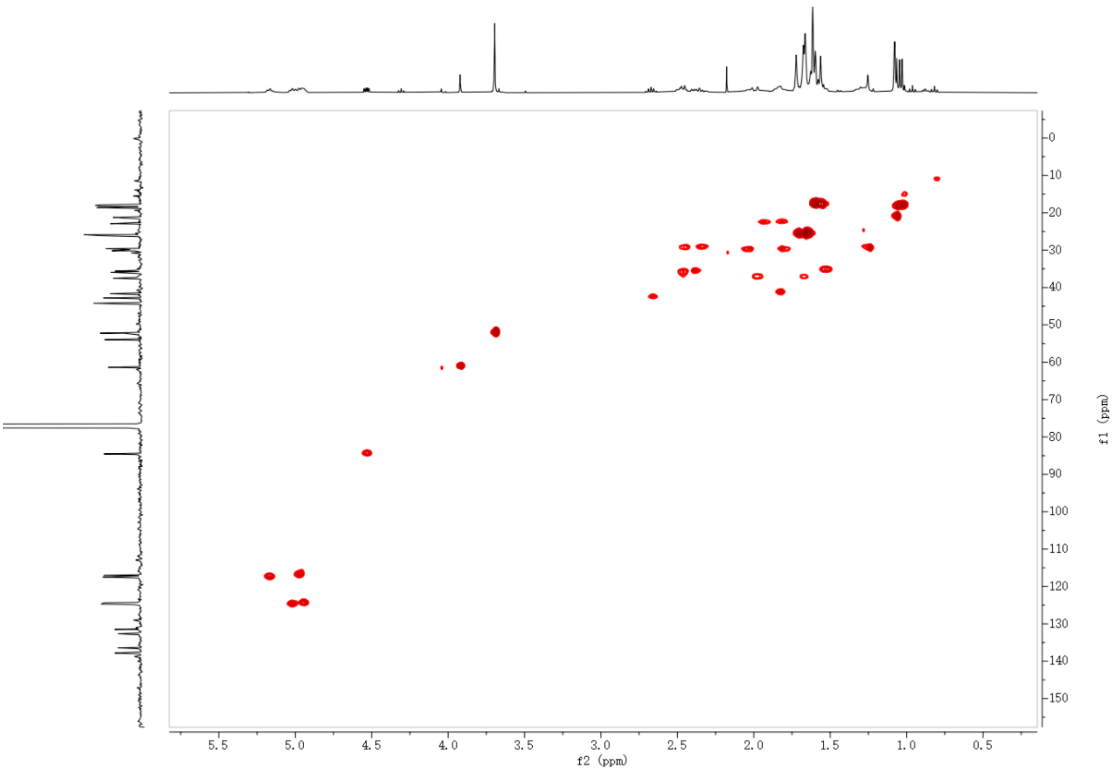

Figure S16. The HMBC spectrum of Hyperforatum B (**2**).

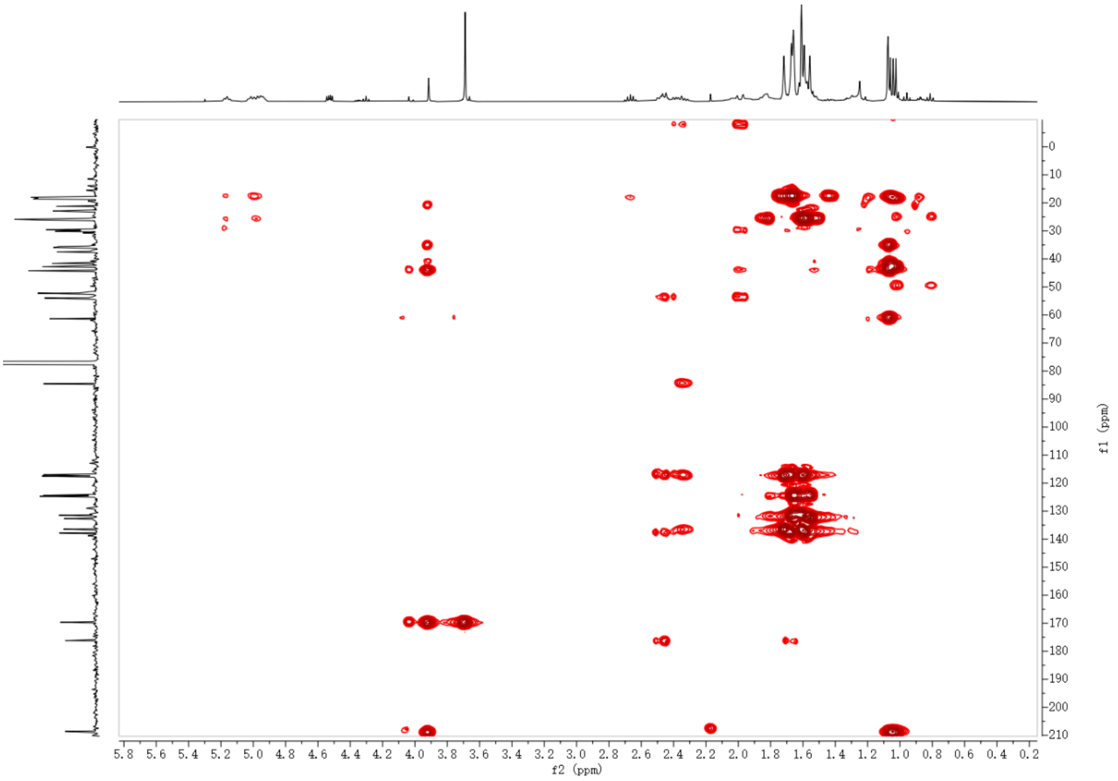

Figure S17. The NOESY spectrum of Hyperforatum B (**2**).

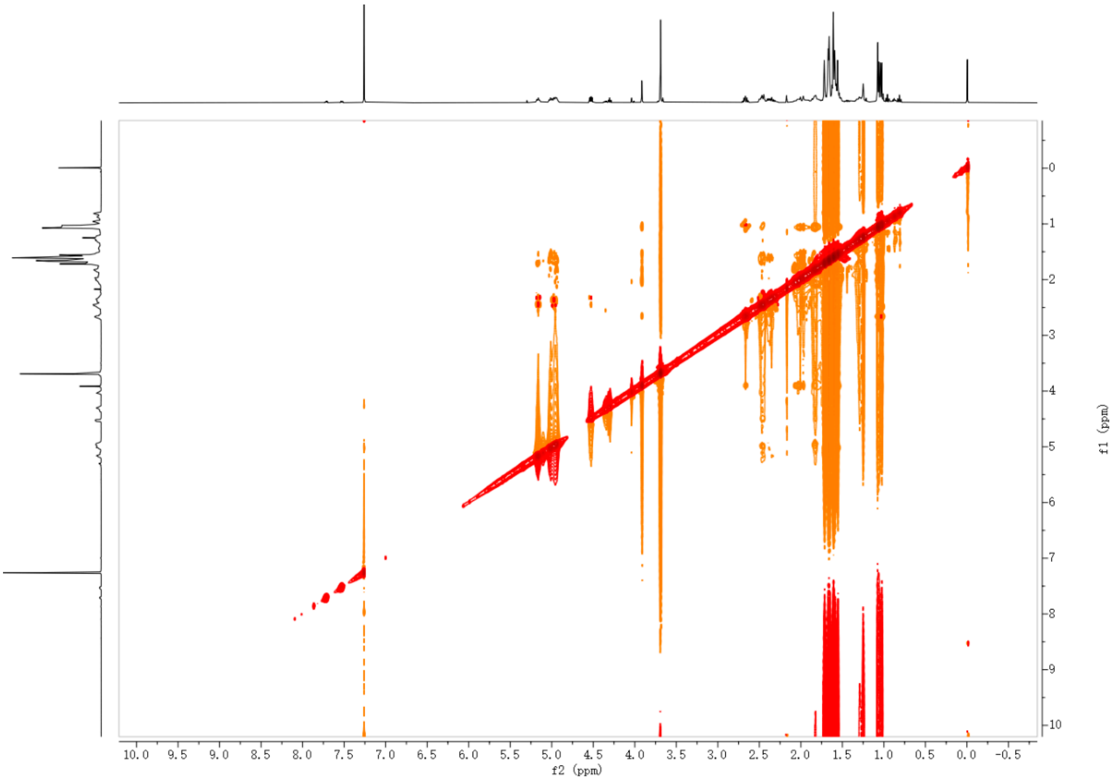

Figure S18. The HRESIMS spectrum of Hyperforatum B (**2**).

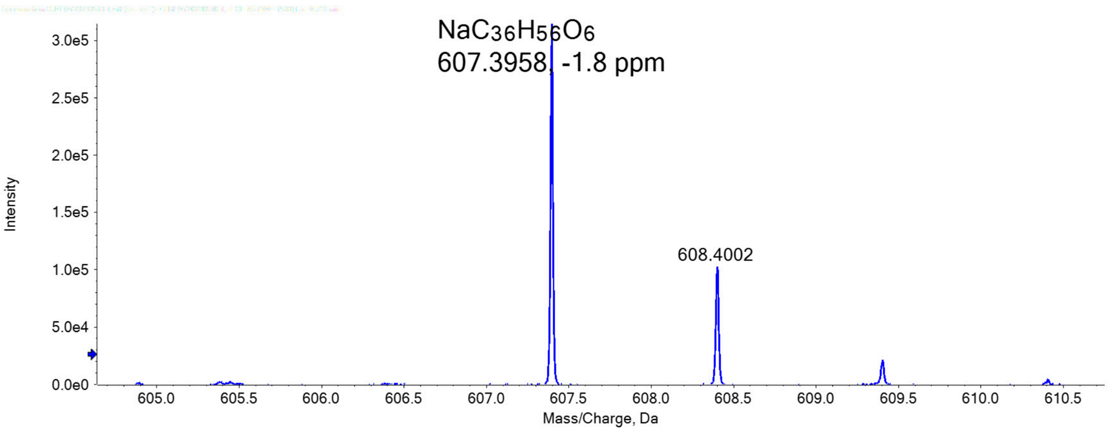

Figure S19. The UV spectrum of Hyperforatum B (**2**).

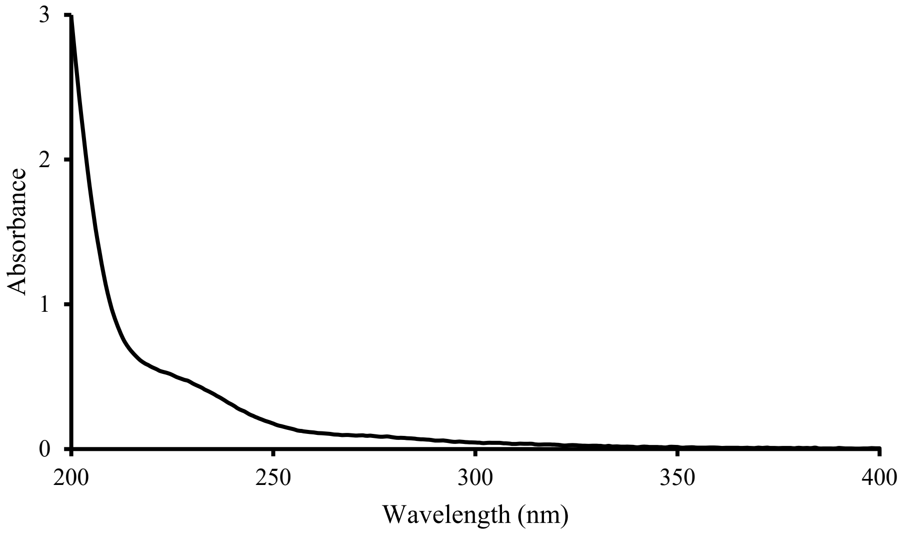

Figure S20. The Experimental ECD spectrum of Hyperforatum B (**2**).

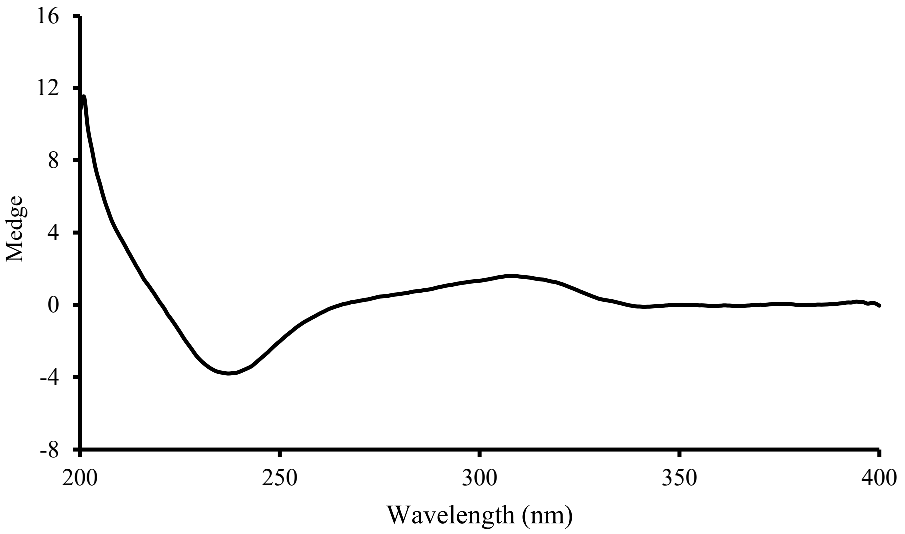

Figure S21. The ^{1}H NMR spectrum of Hyperforatum C (**3**) in $CDCl_3$ (400 MHz).

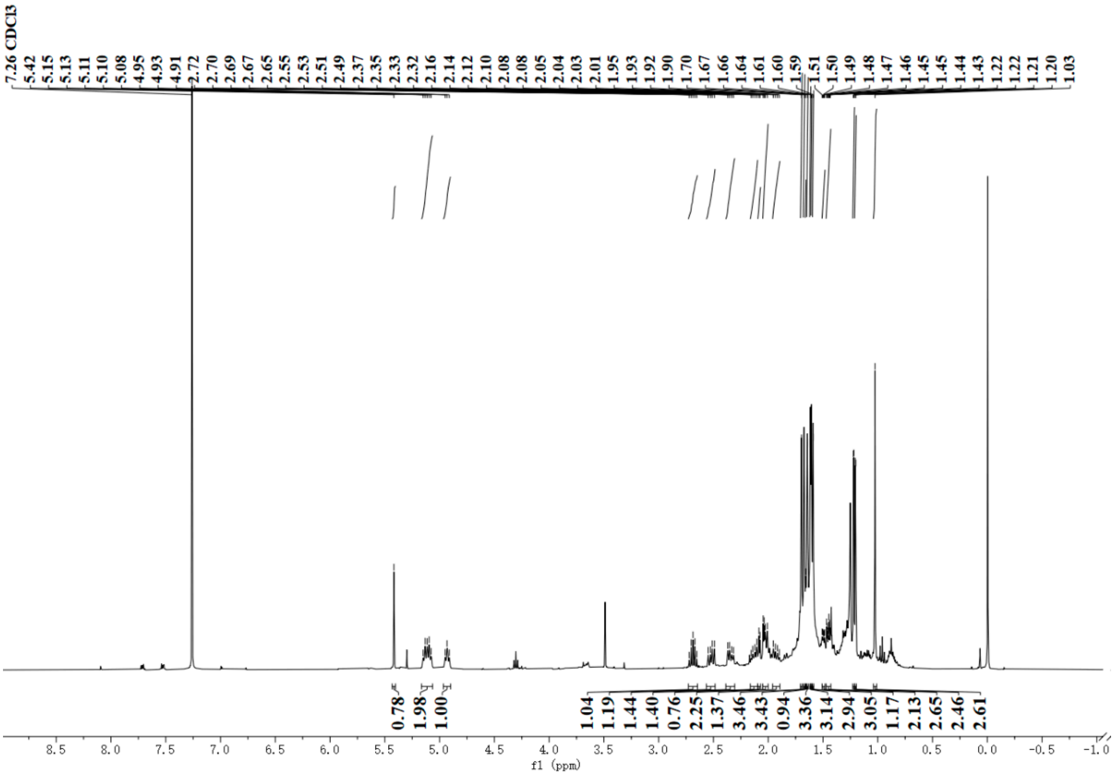

Figure S22. The ^{13}C NMR spectrum of Hyperforatum C (**3**) in $CDCl_3$ (100 MHz).

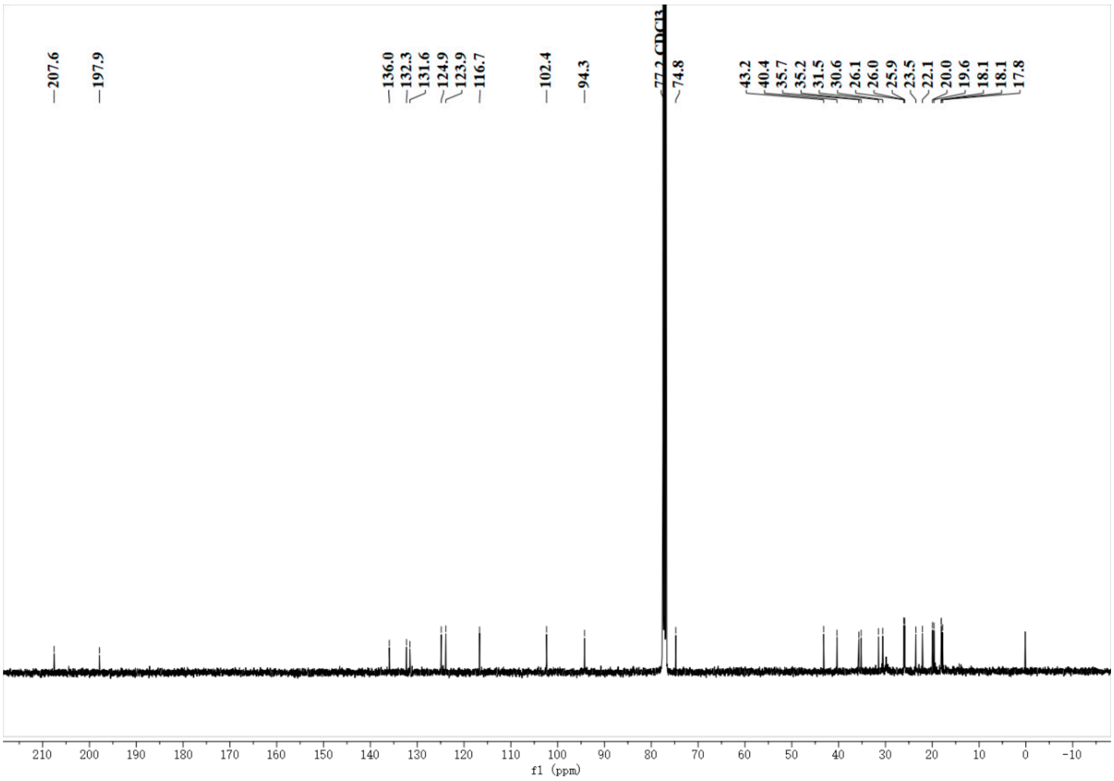

Figure S23. The DEPT 135° spectrum of Hyperforatum C (**3**) in CDCl3.

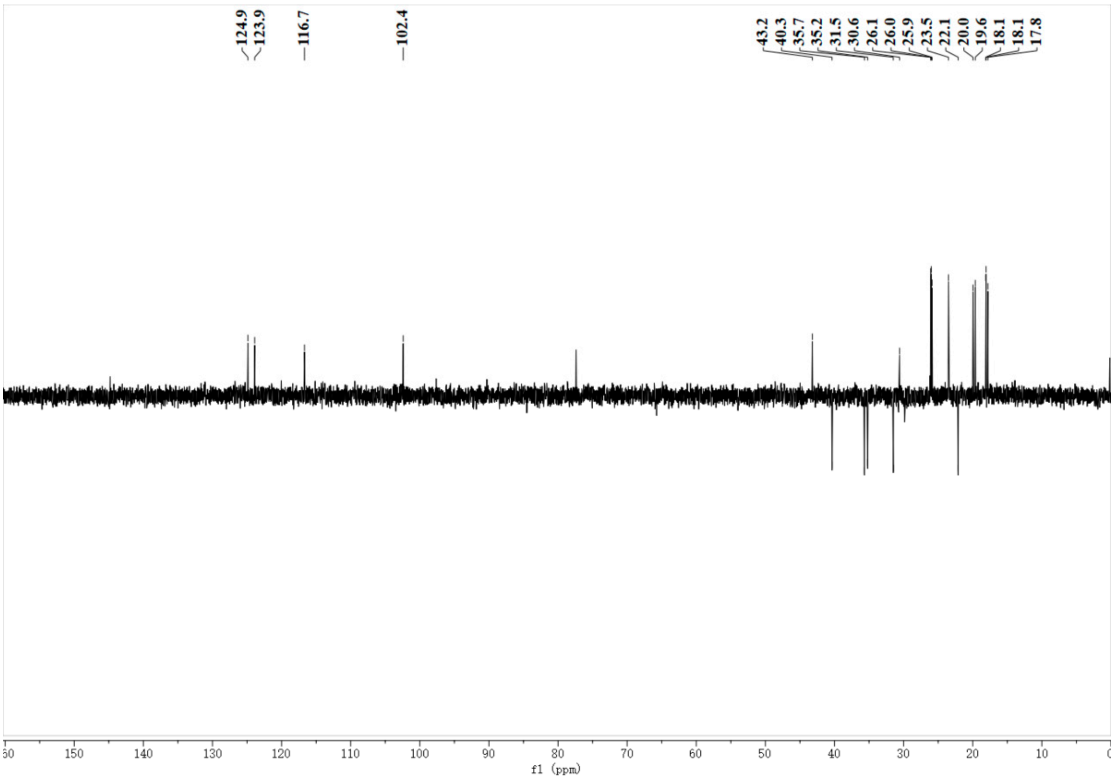

Figure S24. The ^{1}H-^{1}H COSY spectrum of Hyperforatum C (**3**).

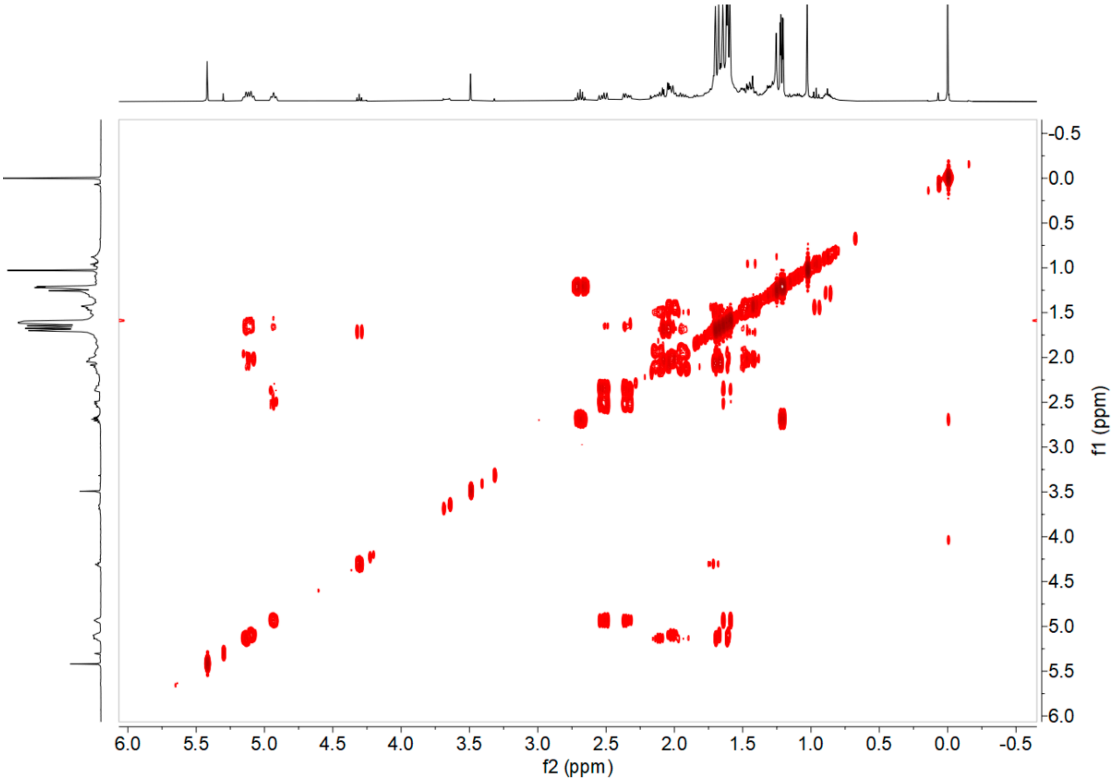

Figure S25. The HSQC spectrum of Hyperforatum C (**3**).

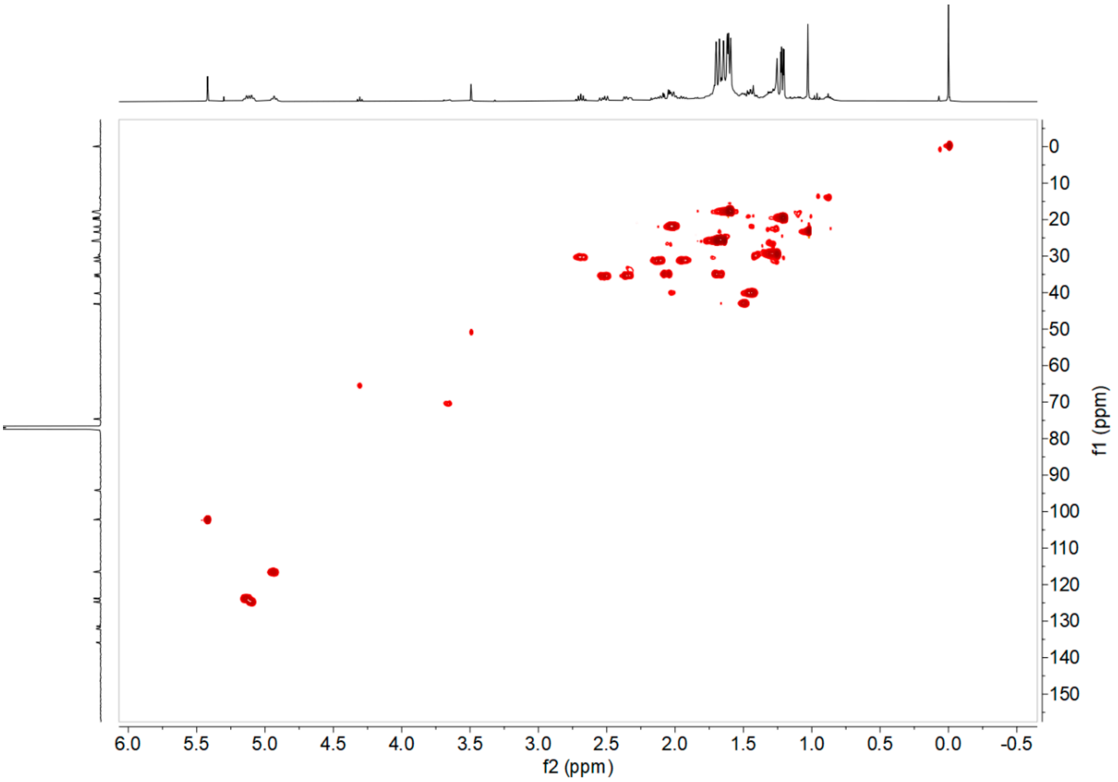

Figure S26. The HMBC spectrum of Hyperforatum C (**3**).

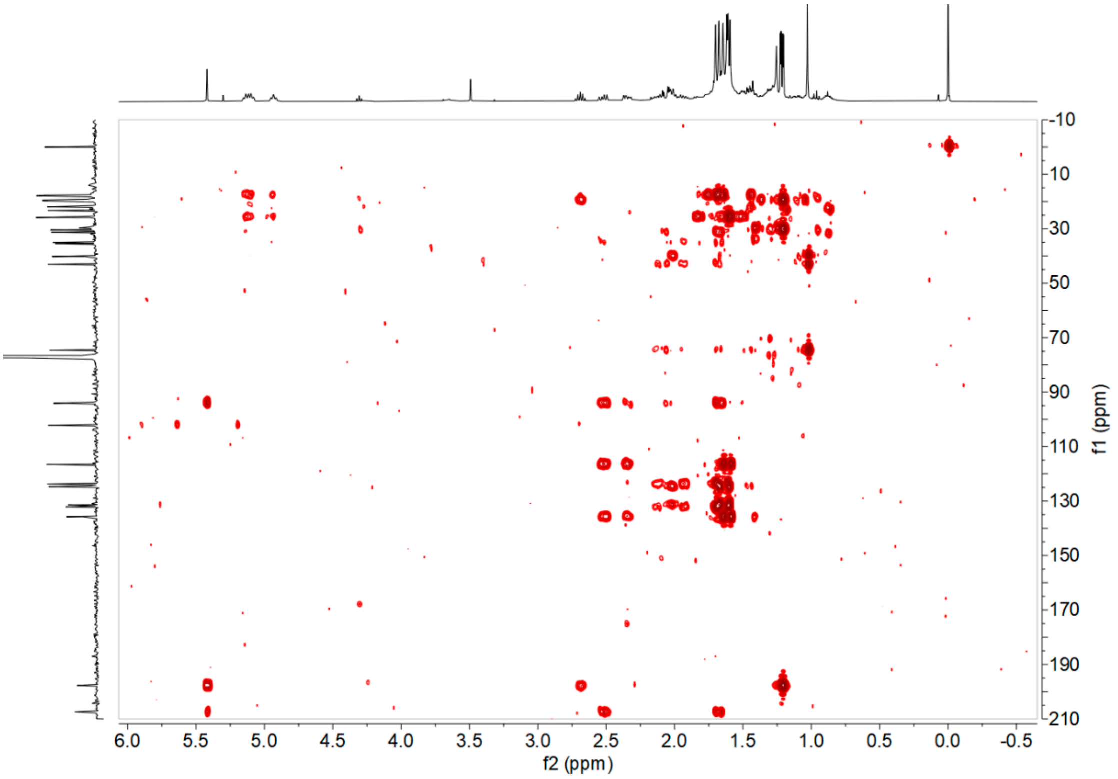

Figure S27. The NOESY spectrum of Hyperforatum C (**3**).

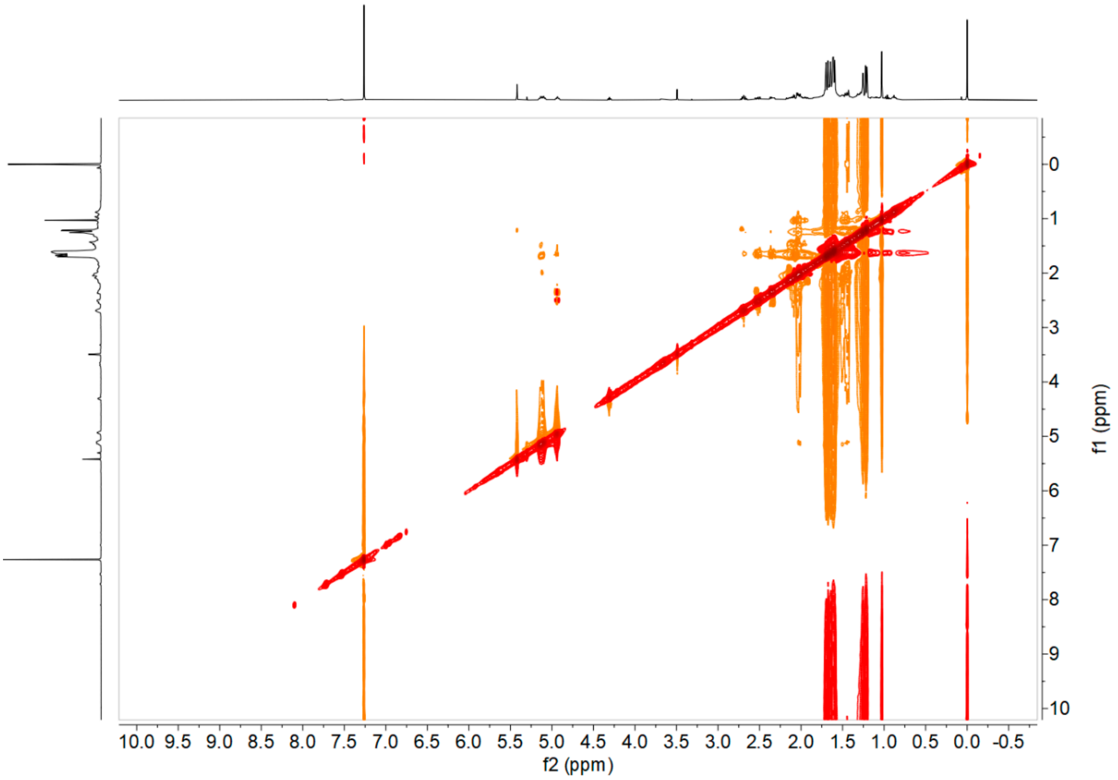

Figure S28. The HRESIMS spectrum of Hyperforatum C (**3**).

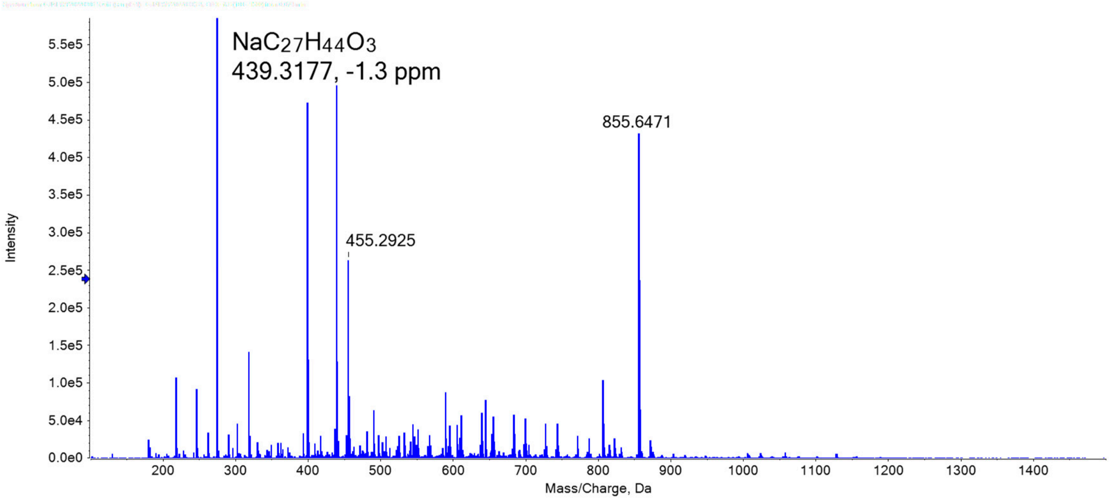

Figure S29. The UV spectrum of Hyperforatum C (**3**).

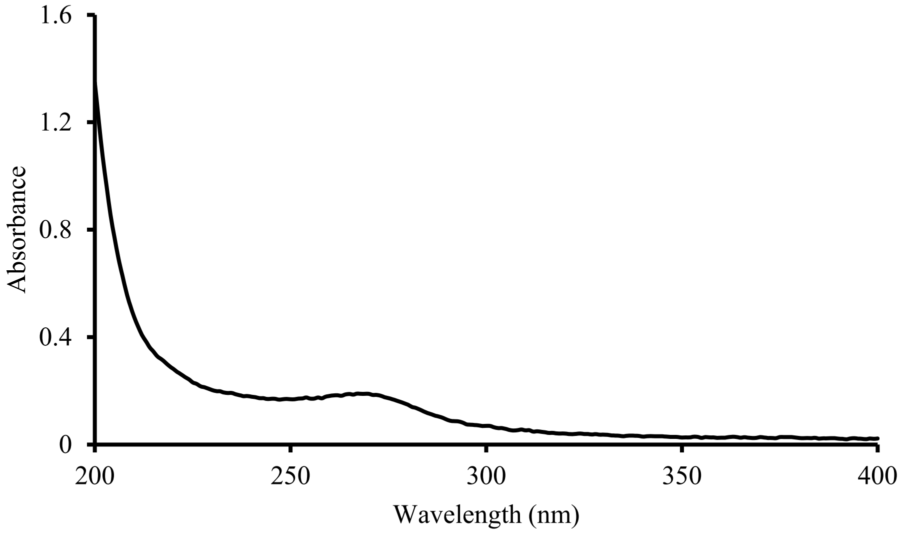

Figure S30. The Experimental ECD spectrum of Hyperforatum C (**3**).

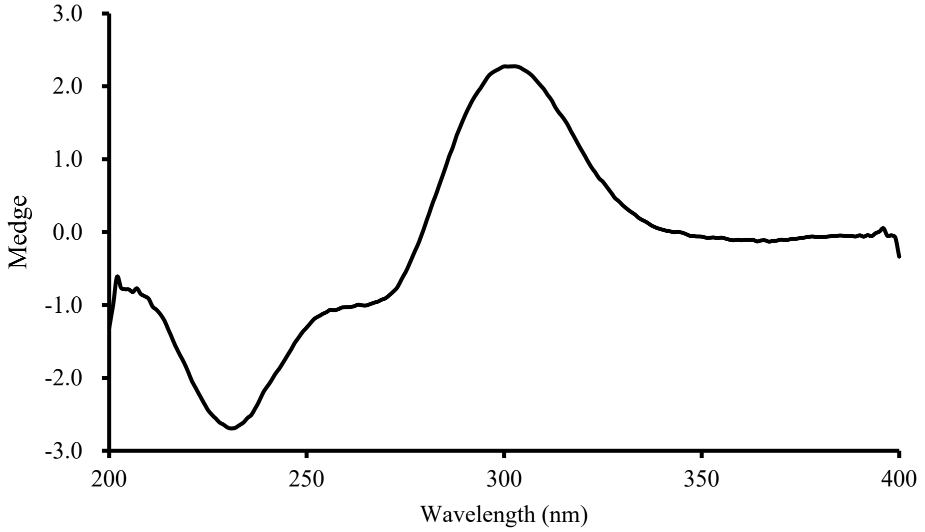

Figure S31. The ^{1}H NMR spectrum of Hyperforatum D (**4**) in $CDCl_3$ (800 MHz).

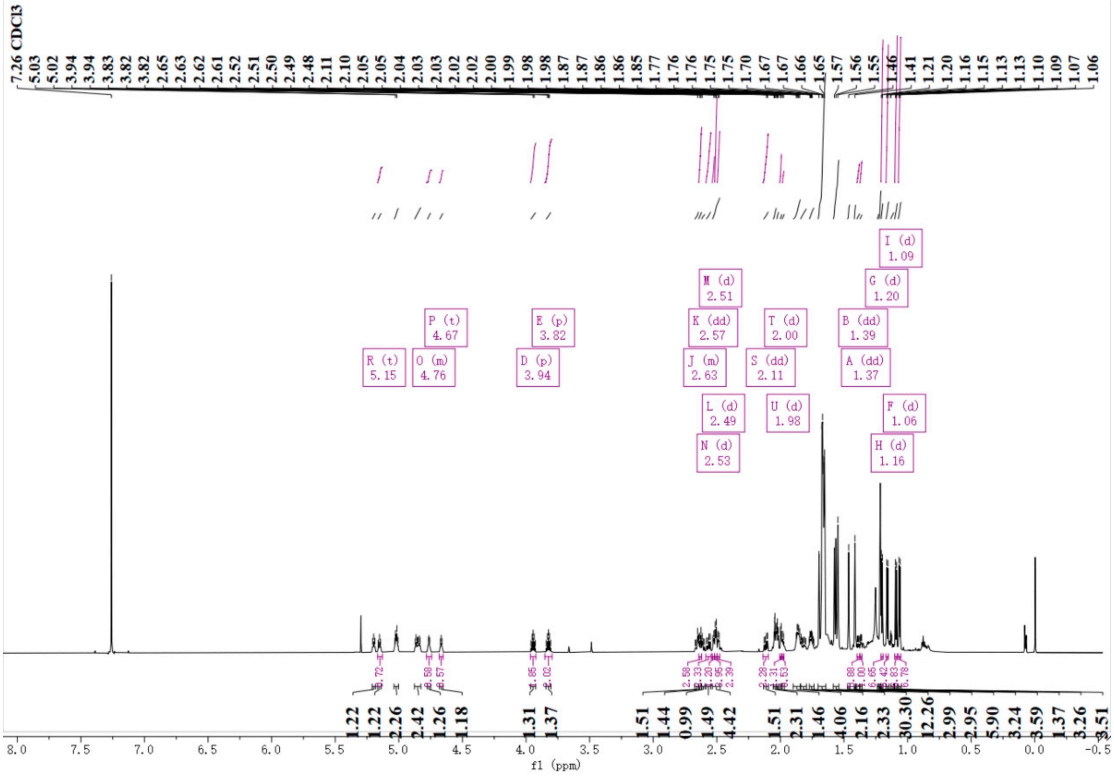

Figure S32. The ^{13}C NMR spectrum of Hyperforatum D (**4**) in $CDCl_3$ (200 MHz).

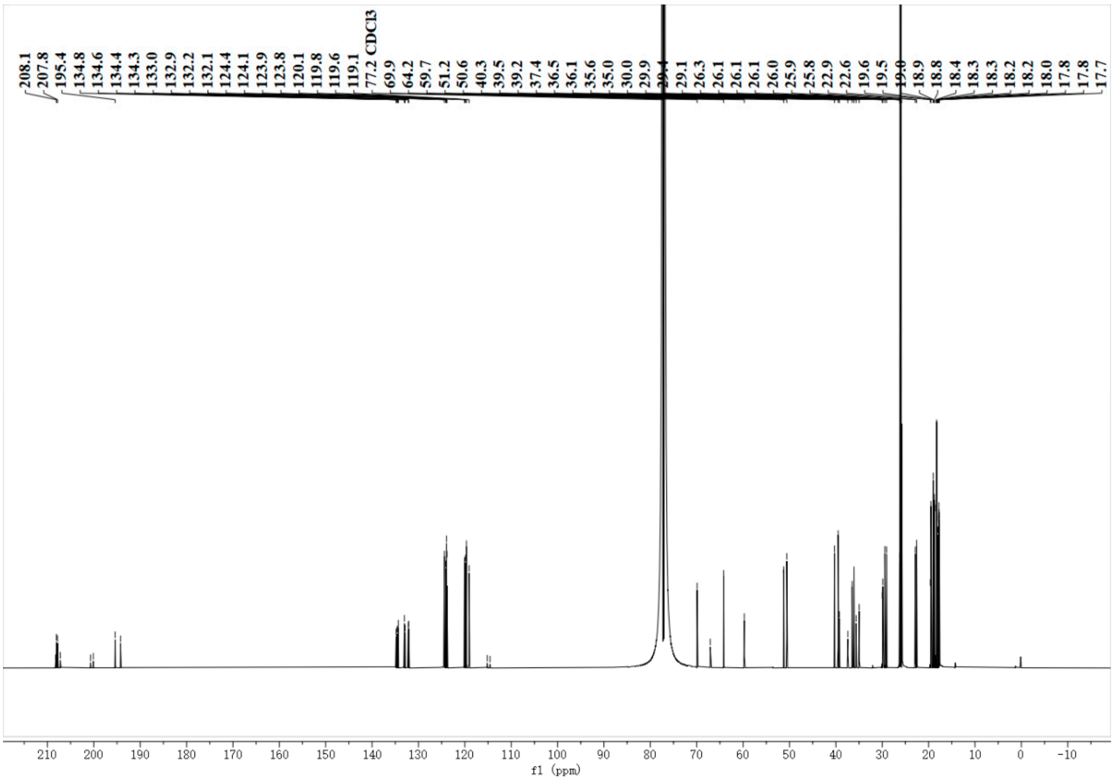

Figure S33. The DEPT 135° spectrum of Hyperforatum D (**4**) in $CDCl_3$.

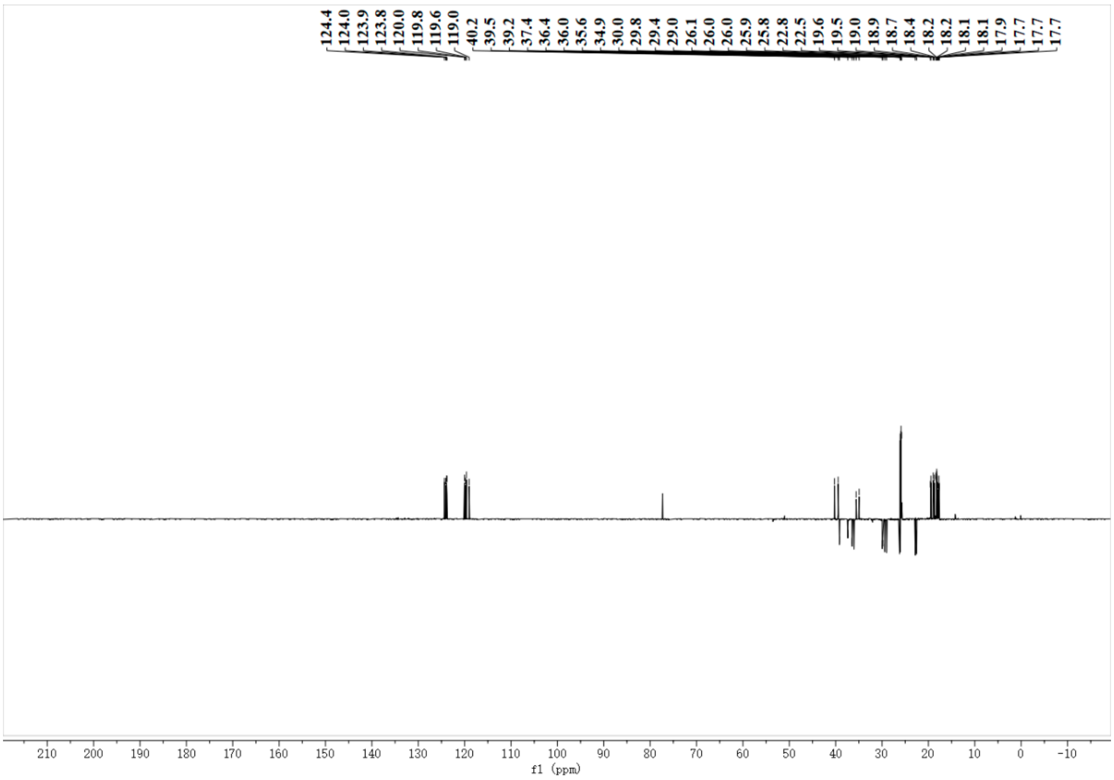

Figure S34. The ^1H-^1H COSY spectrum of Hyperforatum D (**4**).

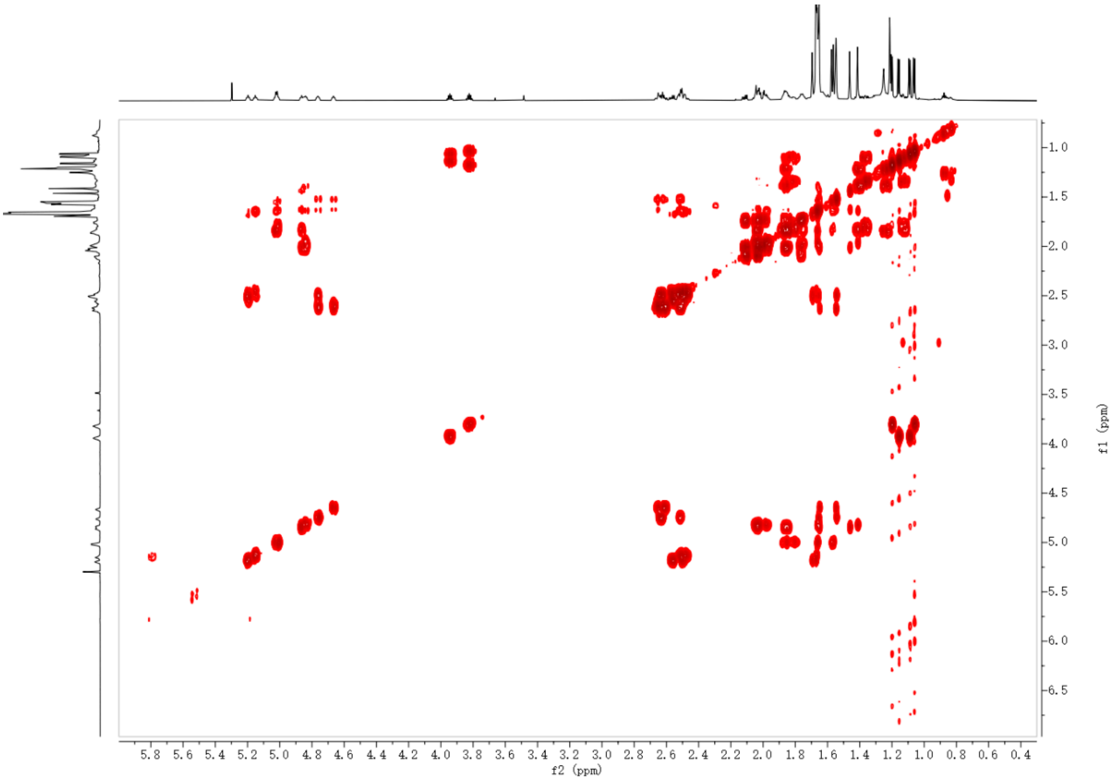

Figure S35. The HSQC spectrum of Hyperforatum D (**4**).

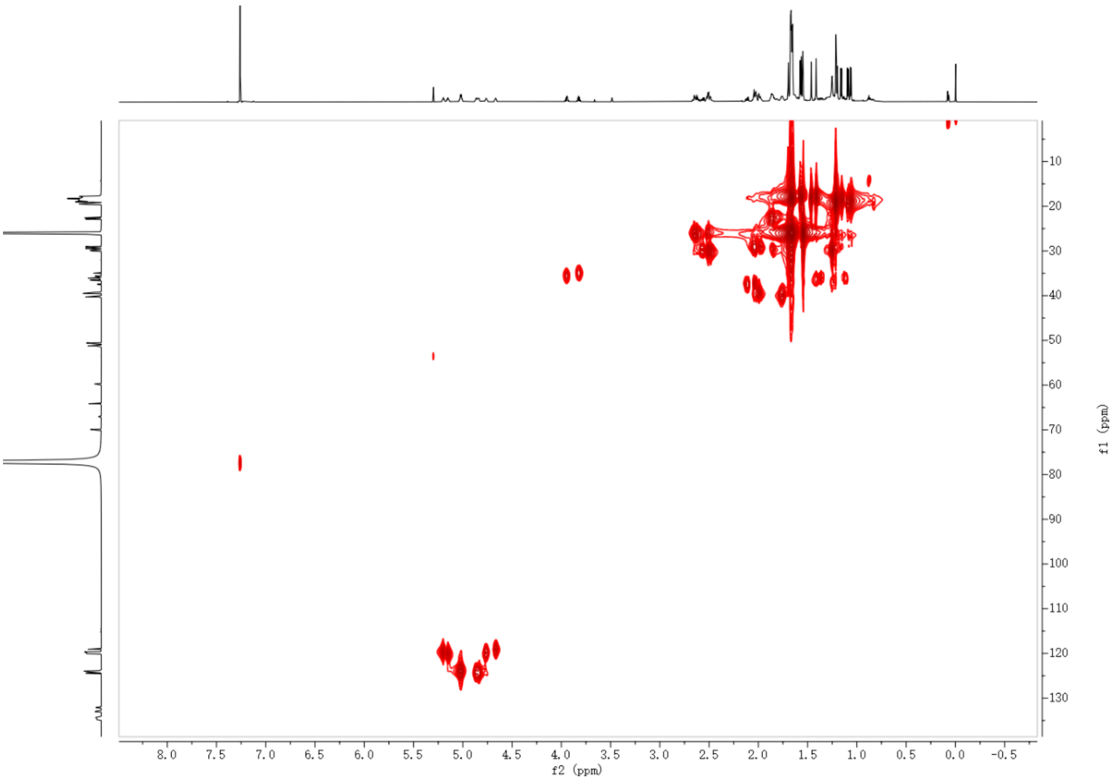

Figure S36. The HMBC spectrum of Hyperforatum D (**4**).

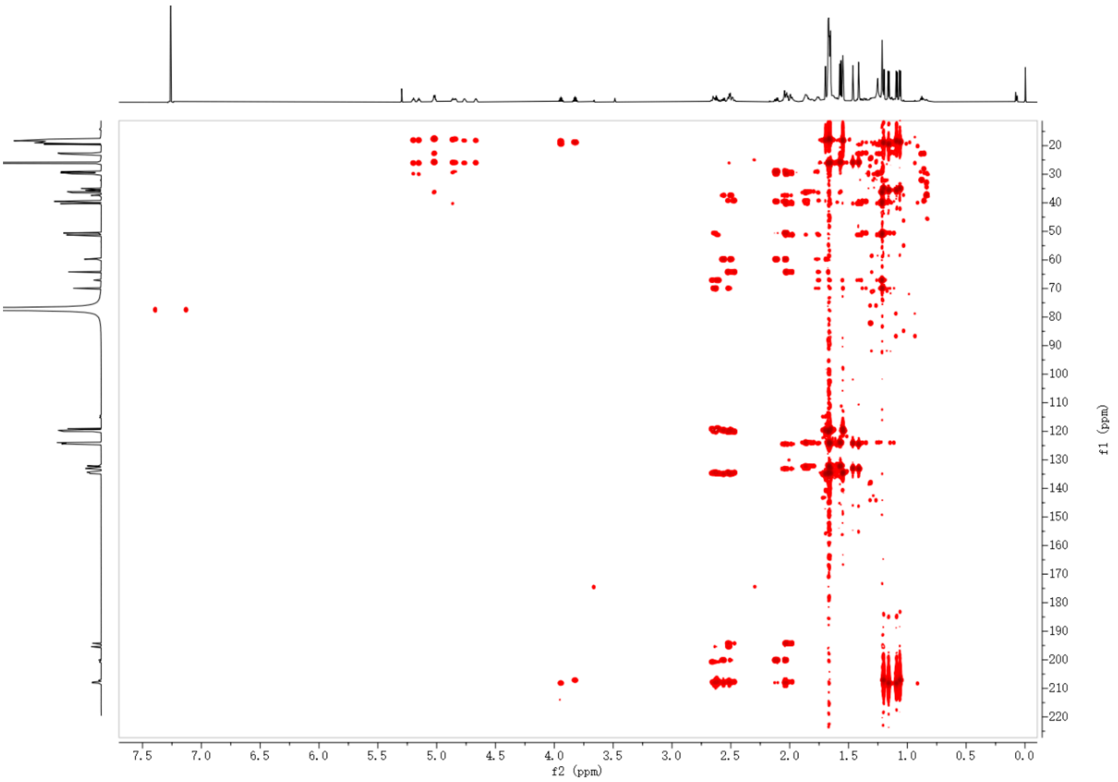

Figure S37. The NOESY spectrum of Hyperforatum D (**4**).

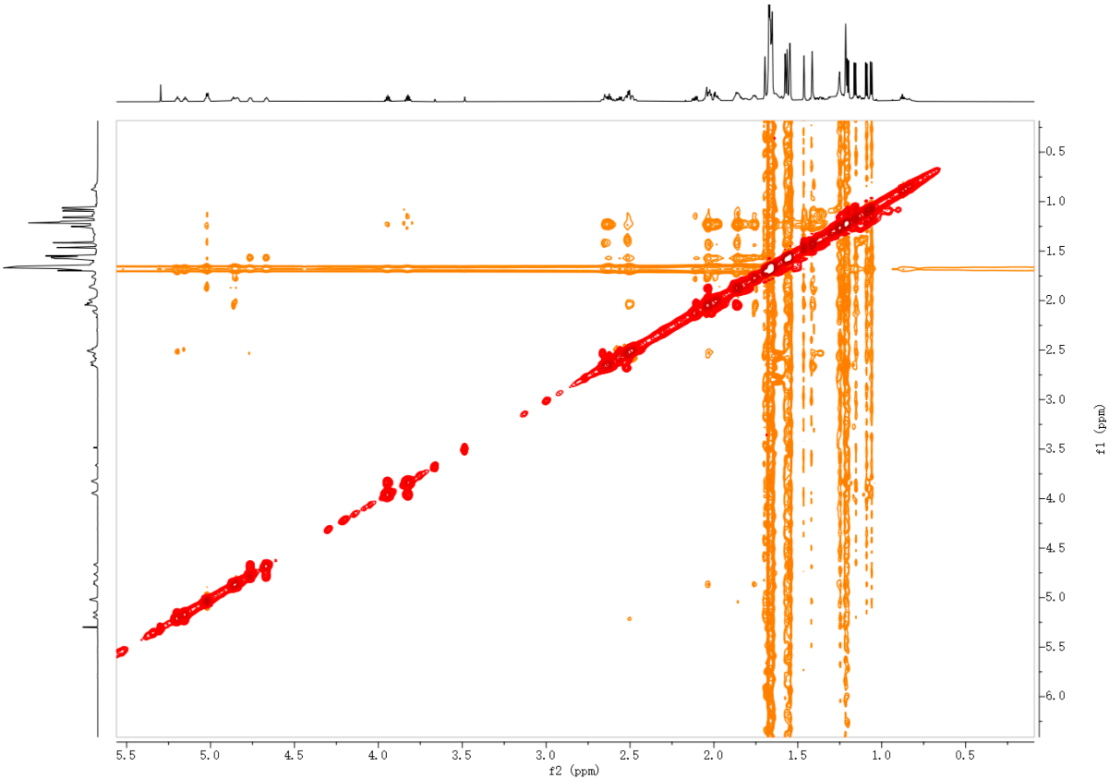

Figure S38. The HRESIMS spectrum of Hyperforatum D (**4**).

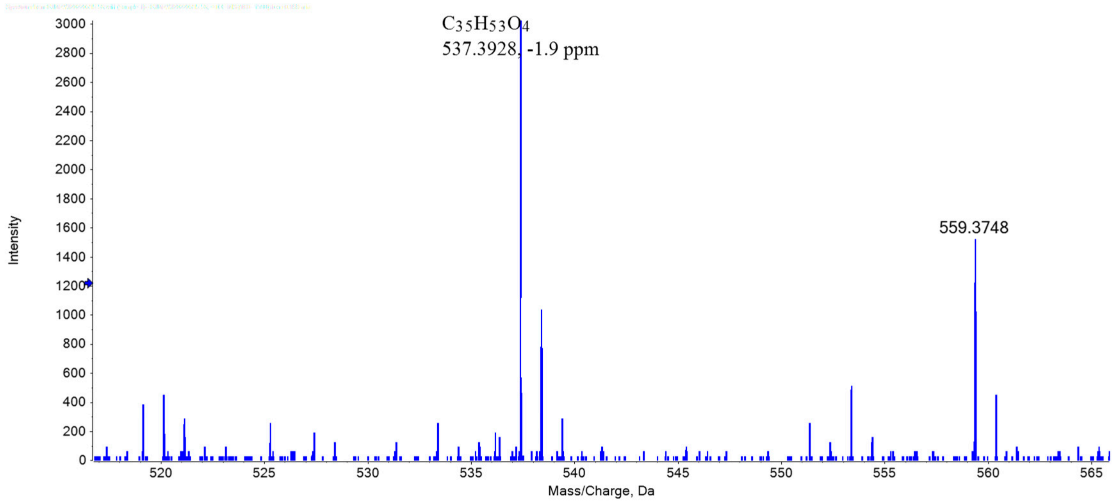

Figure S39. The UV spectrum of Hyperforatum D (**4**).

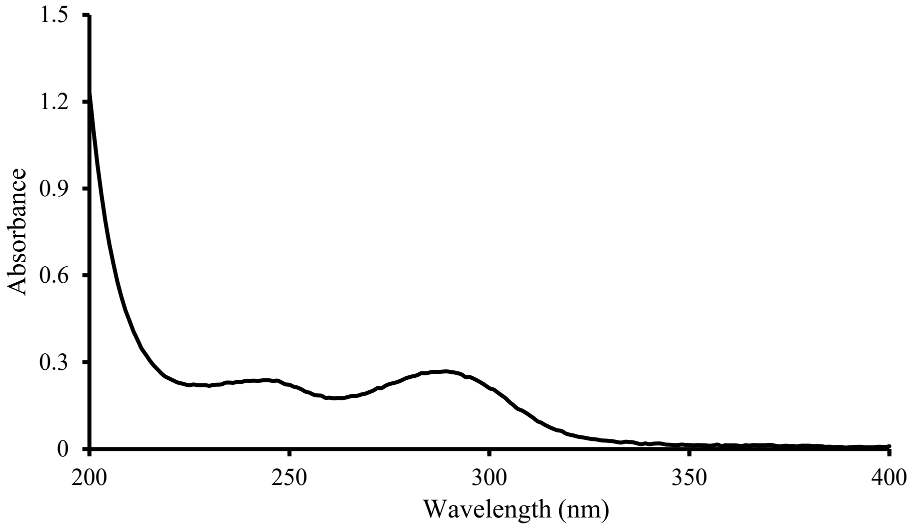

Figure S40. The Experimental ECD spectrum of Hyperforatum D (**4**).

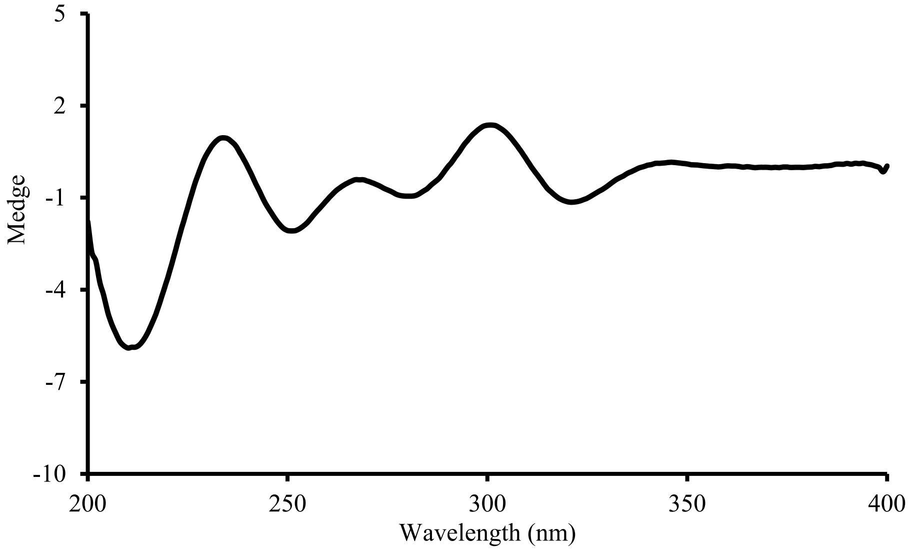

Supplement: Supplementary file 1 [file molecules-29-01756-s001.zip › Supporting information.pdf]
